# Competitive Effects of Cu(II) on the Adsorption of Cd(II), Mn(II), and Zn(II) from Neutral Mine Drainage Using Low-Cost Adsorbents

**DOI:** 10.3390/ma19183997

**Published:** 2026-09-20

**Authors:** Anna Ďuricová, Jarmila Schmidtová, Adrián Biroň, Miroslav Szilágyi, Michal Sečkár, Jozef Salva, Miroslav Vanek, Adam Pochyba, Marián Schwarz, Dagmar Samešová, Juraj Poništ, Eva Výbohová, Darina Veverková

**Affiliations:** 1 Department of Environmental Engineering, Faculty of Ecology and Environmental Sciences, Technical University in Zvolen, T. G. Masaryka 24, 96001 Zvolen, Slovakiaxszilagyi@is.tuzvo.sk (M.S.); schwarz@tuzvo.sk (M.S.);; 2 Department of Mathematics and Descriptive Geometry, Faculty of Wood Sciences and Technology, Technical University in Zvolen, T. G. Masaryka 24, 96001 Zvolen, Slovakia; 3 Earth Science Institute v.v.i., Slovak Academy of Sciences, Ďumbierska 1, 97411 Banská Bystrica, Slovakia; 4 Department of Chemistry and Chemical Technology, Faculty of Wood Sciences and Technology, Technical University in Zvolen, T. G. Masaryka 24, 96001 Zvolen, Slovakia; vybohova@is.tuzvo.sk; 5 Institute of Foreign Languages, Technical University in Zvolen, T. G. Masaryka 24, 96001 Zvolen, Slovakia

**Keywords:** neutral mine drainage, adsorption, low-cost adsorbent

## Abstract

**Highlights:**

- Competitive adsorption of Cu(II), Cd(II), Mn(II), and Zn(II) was evaluated in real mine water.
- Biomass fly ash showed adsorption performance comparable to bentonite and zeolite.
- Cu(II) increased Mn(II) and Zn(II) removal but inhibited Cd(II) adsorption on fly ash.
- Metal competition was governed by electronegativity and ionic radius differences.
- Fly ash represents a promising low-cost adsorbent for mine water treatment.

**Abstract:**

Neutral mine drainage may contain potentially toxic metals even under circum-neutral pH conditions. This study investigated the competitive effect of Cu(II) on the removal of Cd(II), Mn(II), and Zn(II) from real neutral mine drainage using unmodified bentonite, natural zeolite, and biomass-derived fly ash. Batch experiments were conducted for 120 min using 0.5 g of adsorbent per 100 cm^3^ of mine water, with Cu(II) additionally introduced at 20 mg·dm^−3^. Biomass fly ash increased the solution pH to 9.4 and achieved the highest removal efficiencies in the presence of Cu, reaching 98.8% for Cu(II), 99.3% for Mn(II), and 100.0% for Zn(II). In contrast, Cd(II) removal by fly ash decreased from 57.3% without Cu to 0.0% with Cu, whereas Cd(II) removal increased from −199.8% to 100.0% with bentonite and from −224.6% to 38.4% with zeolite. Cu addition also increased Mn(II) removal from −5.1% to 85.6% for bentonite, from −20.7% to 71.4% for zeolite, and from −40.0% to 99.3% for fly ash, while Zn(II) removal increased to 100.0%, 96.8%, and 100.0%, respectively. Two-way analysis of variance confirmed highly significant Cu × adsorbent interactions for Cd(II) (*p* < 0.001), Mn(II) (*p* < 0.001), and Zn(II) (*p* < 0.001). Langmuir analysis gave the highest calculated *q_m_* for Mn(II) with Cu addition: 0.864 mg·g^−1^ for zeolite, 0.754 mg·g^−1^ for fly ash, and 0.720 mg·g^−1^ for bentonite; for fly ash, these values should be interpreted considering simultaneous adsorption and precipitation. No desorption of Cu(II), Mn(II), or Zn(II) was observed after adsorption. Overall, biomass fly ash showed strong potential for Cu(II), Mn(II), and Zn(II) removal from real neutral mine drainage, while the competitive suppression of Cd(II) highlights the importance of multicomponent interactions in practical treatment systems.

## 1. Introduction

The extraction of mineral resources represents an important source of raw materials, while at the same time, it may create environmental pressure related mainly to changes in groundwater quantity and quality. This pressure becomes particularly evident when mining is conducted below the natural groundwater level, inevitably requiring dewatering of the rock environment, which leads to a local to regional decline in groundwater levels [1]. In addition to hydrogeological changes, mine dewatering may also affect the chemical composition of discharged waters. When sulfide minerals present in the exposed rock environment are oxidized, acidic mine drainage (AMD) may be formed, typically characterized by low pH and elevated concentrations of sulfates, iron, and other metals and metalloids. However, in deposits containing a significant proportion of carbonate minerals, such as calcite and dolomite, acidity can be partially neutralized. Under such conditions, mine waters may develop a more neutral character, referred to as neutral mine drainage (NMD), in which concentrations of dissolved pollutants are generally lower than in AMD [2]. Although less acidic, NMD may still contain environmentally relevant concentrations of potentially toxic elements and therefore requires appropriate treatment. The occurrence of NMD has been documented worldwide, including in Slovakia [3,4,5], Zambia [2], Sweden [6], South Korea [7], Italy [8], Brazil [9], and Tunisia [10]. Environmental relevance of NMD is mainly associated with the presence and mobility of dissolved heavy metals.

Removal of heavy metals from mine drainage and other contaminated waters represents an important aspect of environmental protection and public health. Heavy metals are persistent, may bioaccumulate, and can be toxic even at low concentrations. Their presence in aquatic environments can contribute to ecosystem degradation, soil contamination, and subsequent entry into the food chain, posing risks to both humans and biota. Therefore, the development and application of effective wastewater treatment technologies are essential to minimize the environmental impacts associated with mine drainage and other contaminated water streams [11].

Treatment methods used for the removal of heavy metals from wastewater include: adsorption, sedimentation and flocculation, co-precipitation, complexation, uptake by constructed wetlands, oxidation/reduction, chemical precipitation, ion exchange, membrane separation techniques, photocatalysis, solvent extraction, and electro-remediation [12]. Among these methods, adsorption is considered one of the most advantageous and cost-effective techniques [13], mainly due to its high efficiency, technical simplicity, and operation without substantial sludge generation. Activated carbon is widely used in adsorption processes because of its excellent sorption properties; however, its broader application is limited by relatively high costs. For this reason, increasing attention is being directed toward more affordable adsorbent materials that can provide sufficient removal efficiency while reducing the economic demands of the treatment process [14].

Despite the economic advantages, natural adsorbents may exhibit limited removal efficiency, which has led to the development of chemically modified materials. The performance of adsorbents can be improved by chemical modification, which may increase their adsorption capacity to bind to heavy metal ions from solution. This improvement is usually associated with a higher number of active binding sites, enhanced ion-exchange properties, and the formation of new functional groups that support metal capture. Nevertheless, the economic aspect of such modification must also be considered, particularly when evaluating the practical applicability of low-cost adsorbents. The costs of chemicals and modification procedures can substantially increase the overall treatment expenses [15]. This issue is especially important in the treatment of mine drainage, where very large volumes of water are often generated. In such cases, extensive chemical modification of natural low-cost adsorbents may be unrealistic for full-scale industrial application. Therefore, the use of locally available, unmodified natural or waste-derived adsorbents represents a more practical and economically feasible approach for the treatment of heavy metal-contaminated waters [16].

The main objective of this study is to evaluate the effect of variability in Cu(II) concentration on the adsorption efficiency and selectivity of low-cost adsorbents (bentonite, zeolite, and biomass ash) for the removal of Cd(II), Mn(II), and Zn(II) from real neutral mine water. The work focuses on identifying positive and negative adsorption effects induced by competition among metals for active sorption sites while also monitoring the stability of the immobilized elements through assessment of their desorption behavior.

The aim of the research is to verify the competitiveness of waste-derived ash compared to natural sorbents under conditions close to real industrial practice, thereby contributing to the development of efficient and economically sustainable technologies for the treatment of waters contaminated by mining activities.

Another objective was to evaluate the desorption of selected elements from the adsorbents back into the solutions. This release is an expected phenomenon due to the high content of adsorbed elements in the used adsorbents. However, it is necessary to confirm this through empirical results. Desorption resulting from an improperly selected adsorbent can completely negate the positive effect of adsorption. Unmodified adsorbents were used due to the practical requirement to minimize the costs of the adsorption process and to simplify the implementation of the adsorption technology. Cost minimization and technological simplicity are very important requirements for the feasibility of the process, considering the enormous volume of mine drainage generated. The use of real mine drainage and variation in metal concentrations, along with monitoring the desorption of the studied elements from low-cost, unmodified adsorbents into solutions, contributes to expanding knowledge in the scientific field of treatment of waters contaminated by mining activities.

Water contamination by mine drainage is a global environmental problem. Therefore, research should focus on acquiring knowledge on the treatment of such waters under conditions as close to reality as possible. The adsorption process was investigated in neutral mine drainage (as opposed to the predominantly studied acid mine drainage) due to the presence of such waters in Slovakia [17,18]. However, it should be emphasized that the occurrence of neutral mine drainage has, besides Slovakia, been documented worldwide in other countries as well.

The scientific contribution of this study lies in a comprehensive evaluation of adsorption processes under conditions that closely approximate real industrial practice. In contrast to most previous research based on synthetic solutions, this work uses an authentic sample of neutral mine water (NMD) from the “Voznická dedičná štôlňa”. The innovative aspect is particularly the investigation of the interaction effect of copper (Cu) on the sorption of Cd(II), Mn(II), and Zn(II). At the same time, the study introduces an important economic and environmental perspective, as it verifies the competitiveness of waste-derived ash compared to commercial sorbents (bentonite, zeolite), thereby directly supporting the principles of circular economy and waste valorisation.

The chosen research strategy is based on a comparative analysis of three different low-cost materials—natural bentonite, zeolite, and waste biomass ash—with the aim of identifying their limitations in the treatment of multicomponent mine waters. The methodological novelty of the work lies in the transition from simplified model systems to real-world conditions, achieved by using an authentic sample of neutral mine water with controlled variability in Cu(II) concentration. This approach enabled not only the quantification of adsorption capacities but also a deeper understanding of competitive mechanisms and the stability of metal retention through subsequent desorption tests, providing a comprehensive view of the practical applicability of these materials in environmental engineering.

## 2. Materials and Methods

### 2.1. Sampling Locations

**Figure 1:**
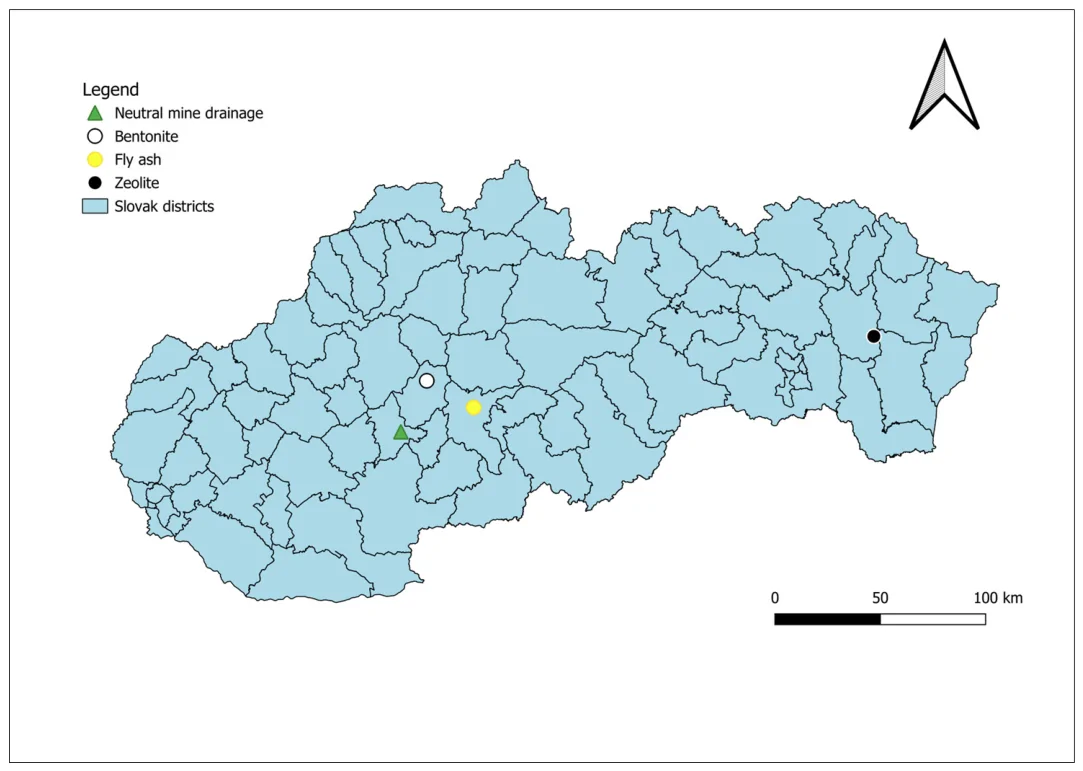
Locations of the sampling sites for the adsorbents and neutral mine drainage (adapted) [19].

Biomass fly ash, natural bentonite, and natural zeolite were used as adsorbent materials. The biomass fly ash originated from a biomass-fired thermal power plant located in Zvolen, Central Slovakia. The facility used clean wood chips as fuel, and the fly ash was recovered from the flue-gas stream by an electrostatic precipitator. The investigated material therefore represented the fine particulate fraction entrained in the flue gas and separated before its release to the atmosphere. After collection, the biomass fly ash was transferred into sealed containers and stored under dry conditions until further use and characterization. Natural bentonite originated from a mineral deposit in the Kopernica area, Slovakia, whereas natural zeolite was obtained from the Nižný Hrabovec deposit in eastern Slovakia. Both materials were used as naturally occurring mineral adsorbents and were subsequently subjected to the physicochemical characterization described in the following section [19]. The geographical locations associated with the origin of all three adsorbent materials are presented in Figure 1.

Neutral mine drainage used in the adsorption experiments was collected from the “Voznická dedičná štôlňa” located in the Štiavnica-Hodruša mining district in Central Slovakia at 48°27′ N and 18°42′ E [20].

Neutral mine drainage samples were collected directly at the monitoring site into clean plastic containers, maintained under cooled conditions during transport, and delivered to the laboratory for subsequent analyses and adsorption experiments. Sampling and sample handling were performed in accordance with STN EN ISO 5667-1:2007 Water Quality—Sampling—Part 1: Guidance on the Design of Sampling Programs and Sampling Techniques [21]. Figure 1 shows the locations of the sampling sites for the adsorbents and neutral mine drainage.

Generated neutral mine drainage represents a relict of mining processes in the area. The estimated total historical production of the mines in the mining district is estimated at 4000 t of Ag and 80 t of Au. Base metal mining, which was active from the 19th century until 1992, yielded approximately 70,000 t of Zn, 55,000 t of Pb, and 8000 t of Cu [20].

### 2.2. Characteristics of NMD

The elemental composition of the adsorbents was determined by ICP-OES (AES-ICP) (atomic emission spectrometry with inductively coupled plasma) method according to the standard ISO 11885 [22], while the concentrations of Cu(II), Cd(II), Mn(II), and Zn(II) in the mine water samples used in the adsorption and desorption experiments were determined by AAS using an AVANTA Σ atomic absorption spectrometer (GBC Scientific, Keysborough, Australia).

The hydrogen ion activity of the samples was measured potentiometrically using a combined electrode in accordance with STN EN ISO 10390 [23]. Measurements were performed with an inoLab pH Level 1 instrument manufactured by WTW Weilheim, Weilheim in Oberbayern, Germany. The measured concentrations of the investigated metals were subsequently compared with the relevant reference and limit values, as presented in Table 1.

For evaluating adsorption from mine drainage, the selection of the target metals is crucial. As a criterion for choosing the adsorbed metals, Cu(II), Zn(II), Cd(II), and Mn(II) were selected because their concentrations exceed the limit values specified in the legislation of the Slovak Republic [24] (Table 2).

### 2.3. Characteristics of Adsorbents

#### 2.3.1. Chemical Composition, Elemental Analysis and BET Characteristic

The metals were determined using the AES-ICP method (atomic emission spectrometry with inductively coupled plasma) according to the ISO 11885 standard [22]. In addition to the chemical composition and elemental analysis, the characterization of the adsorbents was supplemented with the Brunauer–Emmett–Teller (BET) surface area measurement. This approach is widely used to quantify the specific surface area of porous solids and powdered materials. The resulting surface-area values are particularly relevant for materials whose adsorption behavior is influenced by their internal pore structure [25]. The chemical composition, elemental analysis, and BET characteristics of the adsorbents used are presented in Table 3. Nova Station A instrument (Quantachrome) was used to determine the BET surface area of the materials.

#### 2.3.2. ATR-FTIR Spectroscopy

**Figure 2:**
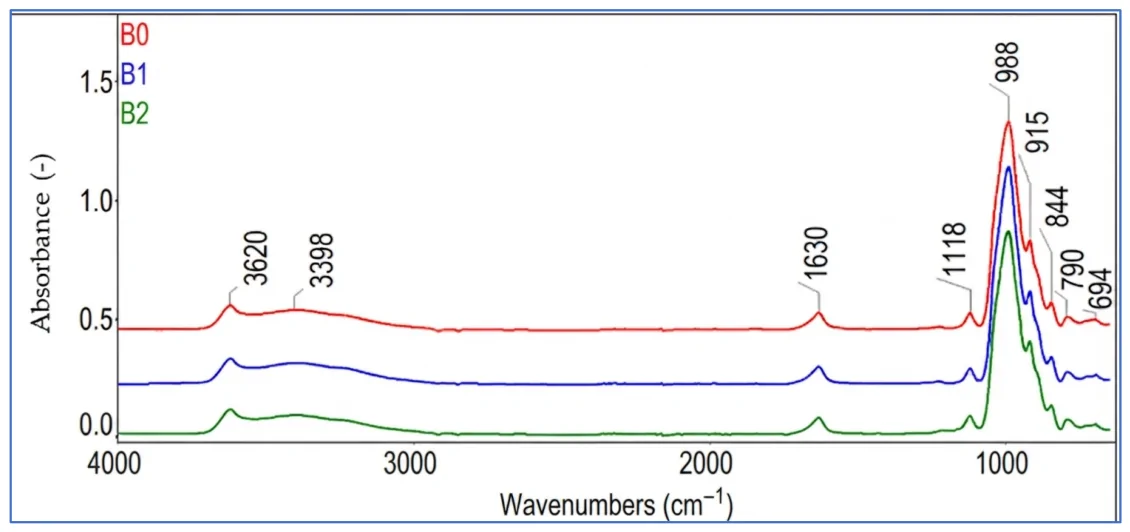
ATR-FTIR spectra of bentonite before adsorption (B0), after adsorption of neutral mine drainage (B1), and after adsorption of mine water with added Cu(II) (B2).

**Figure 3:**
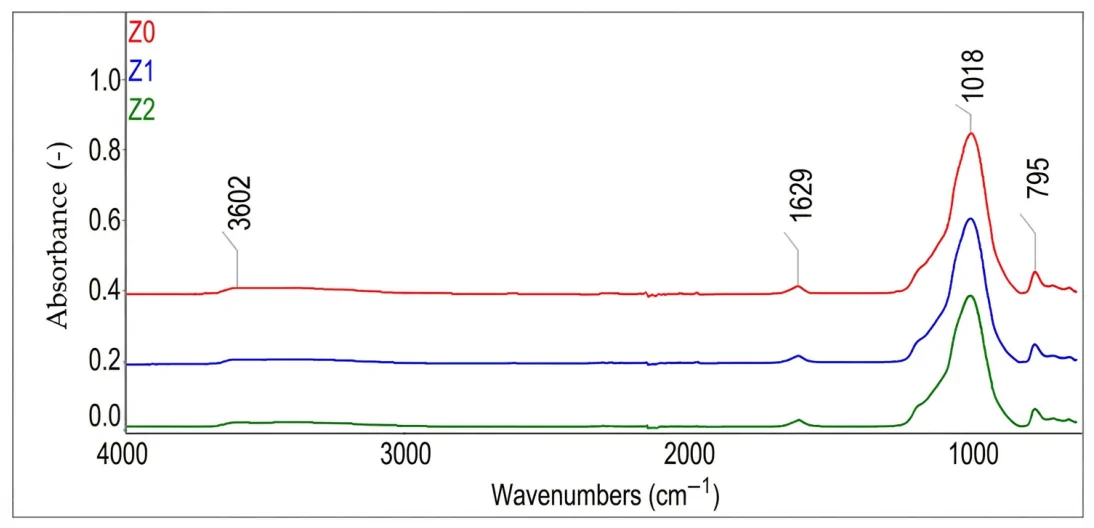
ATR-FTIR spectra of zeolite before adsorption (Z0), after adsorption of neutral mine drainage (Z1), and after adsorption of mine water with added Cu(II) (Z2).

**Figure 4:**
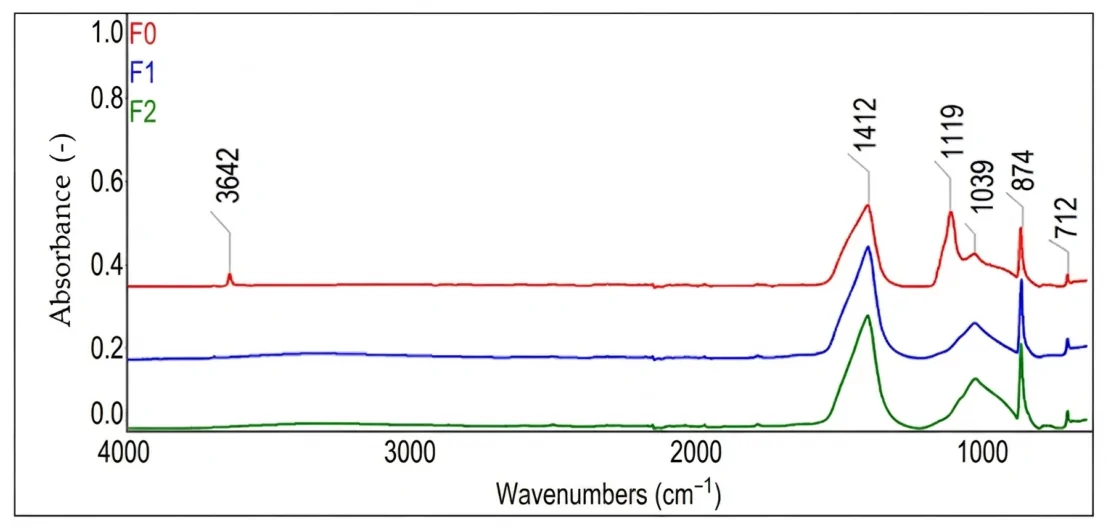
ATR-FTIR spectra of fly ash before adsorption (F0), after adsorption of neutral mine drainage (F1), and after adsorption of mine water with added Cu(II) (F2).

FTIR analysis of the adsorbent materials (bentonite, zeolite, fly ash) was carried out using a Nicolet iS 10 FTIR spectrometer (Thermo Fisher Scientific, Madison, WI, USA) with the attenuated total reflectance (ATR) technique. Spectra were collected using a diamond crystal in the wavenumber range of 4000–650 cm^−1^. For each sample, 32 scans were recorded at a resolution of 4 cm^−1^. The obtained spectra were processed using OMNIC 9 spectroscopic software (Figure 2, Figure 3 and Figure 4). In Figure 2, Figure 3 and Figure 4, the individual spectra have been shifted vertically to improve visual clarity.

In the ATR-FTIR spectrum of raw bentonite (Figure 2), the presence of hydrogen-bonded water is evidenced by a broad band at 3398 cm^−1^, corresponding to O–H stretching vibrations, as well as by the absorption band at 1630 cm^−1^, attributed to the bending vibrations of water [26]. Furthermore, the presence of structural Al–OH groups is confirmed by bands at 3620 cm^−1^ assigned to O–H stretching vibrations and at 915 cm^−1^ corresponding to O–H bending vibrations [27,28]. The relative intensity of both bands with respect to the band at 988 cm^−1^ slightly increases in the B2 spectrum. The absorption band at 988 cm^−1^ is the most intense band in all three bentonite spectra and is attributed to Si–O bending vibrations [29].

In the zeolite spectra (Figure 4), absorption bands of bound water (at 3602 and 1629 cm^−1^), asymmetric stretching vibrations of the Si–O–Si and Si–O–Al bridge bonds at 1018 cm^−1^, and symmetric stretching vibrations of the Si–O–Si bridge bonds at 795 cm^−1^ can be observed [30].

After the adsorption of neutral mine drainage and mine water with added Cu(II), the maximum of the main absorption band shifts toward higher wavenumbers, from 1018 cm^−1^ to 1022 cm^−1^. This shift suggests an increase in the Si/Al ratio of the zeolite structure, likely due to dealumination [31].

In the spectrum of the original fly ash sample (Figure 4), an absorption band of isolated –OH groups can be observed at 3642 cm^−1^; however, this band is no longer present in the spectra of the samples after adsorption. Similarly, the band at 1119 cm^−1^ also disappears after adsorption. This peak can be assigned to the Si–O–Si asymmetric stretching vibrations of silica [32]. Its absence in the spectra of samples F1 and F2 suggests that, in both cases, silica nanoparticles were released (leached) into the solution. SiO_2_ occurs in higher amounts in fly ash from wood residue or contaminated/demolished wood as well as from pine chips [33].

The absorption bands at 1412 and 874 cm^−1^ are present in all three spectra. They indicate the presence of calcite [34], which is usually one of the main components of fly ash from wood biomass [33]. A pronounced and relatively stable absorption band is also observed at 1039 cm^−1^, corresponding to the asymmetric stretching vibrations of Si–O–Al [35]. The absorption band at 712 cm^−1^ may be attributed to metal–oxygen stretching vibrations [34].

#### 2.3.3. Point of Zero Charge (PZC) Determination

The point of zero charge (PZC) represents the pH value at which the net electrical charge on the surface of adsorbent particles is zero. At this pH, the surface is not necessarily completely free of electrical charges; rather, the total amounts of positive and negative surface charges are balanced. Determination of the PZC provides important information about the surface properties of adsorbents and can contribute to the interpretation of electrostatic interactions between the adsorbent surface and adsorbed ions.

The PZC was determined using the ionic strength variation method. For each adsorbent, three sets of seven solutions with different initial pH values were prepared by adjusting the pH using HCl and NaOH solutions. Subsequently, 1 g of adsorbent was added to each solution. The suspensions were shaken for 1 h on a horizontal shaker at a frequency of 130 s^−1^, after which the pH in water, pH(H_2_O), was determined. Subsequently, 1 mL of KCl solutions with concentrations of 0.1, 1.0, and 2.0 mol·L^−1^ was added to the respective sets, resulting in three different ionic strengths. The suspensions were shaken again for 30 min, after which the pH in KCl solution, pH(KCl), was determined. The difference between these values was calculated as ΔpH = pH(H_2_O) − pH(KCl). The PZC value was then determined graphically as the intersection of the ΔpH = f[pH(H_2_O)] curve with the *x*-axis [36].

#### 2.3.4. X-Ray Powder Diffraction

The mineralogical composition of the investigated materials was characterized by X-ray powder diffraction (XRPD). For quantitative analysis, 2.0000 g of each sample was combined with 0.5000 g of Al_2_O_3_ internal standard (AL-OX-03-P, nominal particle size 3–4 µm; American Elements Corp., Los Angeles, CA, USA). The mixture was suspended in 4 mL of ethanol and homogenized for 5 min in a McCrone Micronizing Mill (Retsch GmbH, Haan, Germany) equipped with zirconia grinding elements. After milling, the suspension was dried overnight at 75 °C. To improve particle randomization and minimize preferred orientation effects during diffraction measurements, the dried material was resuspended in water and subsequently spray-dried using a Spray Drying Kit developed by The James Hutton Institute, Aberdeen, Scotland [37].

XRPD measurements were performed using a Bruker D8 Advance diffractometer (Bruker AXS GmbH, Karlsruhe, Germany) equipped with a position-sensitive SSD160 detector (Bruker AXS GmbH, Karlsruhe, Germany) operated in one-dimensional mode. CuKα radiation, generated at 40 kV and 40 mA and filtered using a β-filter, was employed as the X-ray source. The optical configuration included a 0.3° divergence slit, a 6 mm beam mask, a detector opening of 0.4984°, and 2.5° primary and secondary Soller slits. Diffraction data were collected over the angular range of 3–70° 2θ with an increment of 0.01948° 2θ and a counting time of 2 s per step. The powdered samples were measured in top-loading sample holders.

The recorded diffraction patterns were processed using DIFFRAC.EVA software Version 4.2.1.10 [38], and crystalline phases were identified by comparison with reference patterns contained in the PDF-2/2010 database of the International Centre for Diffraction Data. Quantitative phase analysis was subsequently carried out by the Rietveld method using TOPAS 5 software and the fundamental parameters approach, with Al_2_O_3_ serving as the internal standard [39,40].

The structural models used as initial inputs for Rietveld refinement were adopted from published crystallographic data. These included arcanite according to McGinnety [41], calcite according to Sitepu et al. [42], hydroxylapatite according to Hughes et al. [43], periclase according to Hazen [44], albite representing the plagioclase phase according to Gualtieri [45], portlandite according to Desgranges et al. [46], and quartz according to Antao et al. [47]. During refinement, the fitted parameters included the radiation emission profile, scale factors, polynomial background, goniometer zero-point correction, sample displacement, lattice parameters, and, where appropriate for the major phases, atomic coordinates. Atomic occupancies and isotropic displacement parameters were not refined and remained fixed at the values given in the respective structural models.

For samples containing substantial proportions of smectite or clinoptilolite, quantitative mineralogical evaluation was additionally performed using RockJock v11. This method applies full-pattern summation together with mineral intensity factors to estimate phase abundances in complex mineral mixtures [48,49].

#### 2.3.5. Microscopic Analysis of the Adsorbents

**Figure 5:**
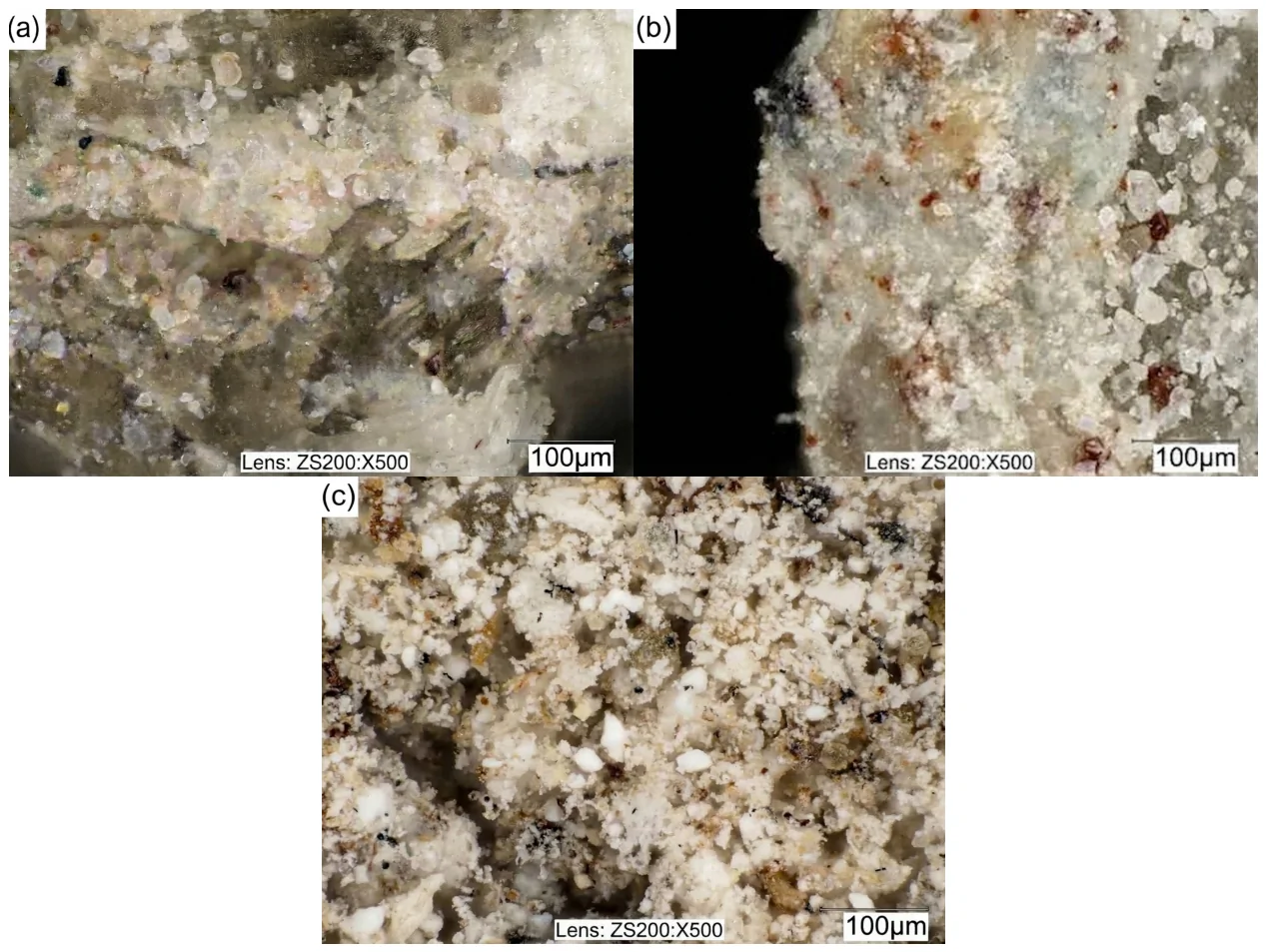
(**a**–**c**) Crystal size in microscopic images for bentonite at magnifications of 500×, for zeolite at magnifications of 500×, and for fly ash at magnifications of 500×. (**a**) Bentonite, displaying a dense, semi-translucent matrix interwoven with fine mineral aggregates and localized veinlets. (**b**) Zeolite, characterized by a distinct grain boundary with well-defined crystalline facets, porous surface coatings, and reddish-brown mineral inclusions. (**c**) Fly ash, exhibiting a highly porous, irregular agglomerate structure composed of fine, loosely bound whitish particles and unburned carbon specks. Scale bars represent 100 µm.

The microscopic images of the crystal sizes of bentonite, zeolite, and fly ash were obtained using a VHX-7000 digital microscope (Keyence Corporation, Osaka, Japan) at magnifications of 500×, as shown in Figure 5a–c.

Microscopic analysis at 500× magnification revealed distinct morphological features across the three investigated samples (Figure 5a–c). As shown in Panel (a), the bentonite displays a cohesive, semi-translucent silicate matrix dominated by fine-grained clay aggregates, with a compact intercrystalline structure interspersed with thin mineral veinlets and minor dark accessory inclusions. In contrast, Panel (b) demonstrates the zeolite sample, which exhibits a distinct interfacial transition between the solid mineral grain and the surrounding void space, characterized by sharply defined crystal faces, fine porous surface coatings, and scattered reddish-brown iron-bearing inclusions. Finally, Panel (c) shows the fly ash, which presents a highly heterogeneous and porous agglomerate texture consisting of fine, irregular whitish particles bound in an open framework, with noticeable dark specks corresponding to unburned carbon and mineral impurities.

### 2.4. Course of the Adsorption Process

The introduction of biomass ash into the system resulted in a sharp increase in the pH of the neutral mine drainage to 9.4, whereas the other tested adsorbents did not affect pH stability. This shift toward alkaline conditions suggests that the removal of the investigated heavy metals may have been partially governed by precipitation processes. Nevertheless, although no macroscopically visible sludge formation was observed during the experiments and all samples were subjected to rigorous filtration through a 5 μm membrane prior to analysis, a minor contribution of surface precipitation or co-precipitation cannot be excluded. The partial removal of metals through precipitation mechanisms is a well-documented phenomenon that may occur over a range of pH values depending on the multielement composition of the solution matrix [50,51]. Previous studies investigating metal adsorption using ash have reported that the increase in solution pH with contact time promoted the removal of metal ions primarily through precipitation, co-precipitation, and adsorption [52].

The volume of each sample in the flasks was 100 cm^3^, and the mass of the added adsorbent was 0.5 g. In experiments without the addition of Cd(II), the adsorbents were added to the unspiked mine water, in which the concentrations of all four target metals had been measured. After equilibrium was reached (120 min of the adsorption process), the concentrations of all four metals were measured again on all adsorbents. Subsequently, the influence of mutual interactions between the metals on the adsorption by bentonite, zeolite, and fly ash was observed. Next, a mine water solution with the addition of Cu(II) at a concentration of 20 mg·dm^−3^ was prepared, and the experiments were repeated in the same manner. After the addition of Cu(II), the effect of the excess Cu(II) on the adsorption of metals onto bentonite, zeolite, and fly ash was monitored. After 120 min of adsorption, the solutions were filtered, and the filtrates were analyzed for the target metals. Adsorption for each solution–adsorbent combination was repeated five times. The monitoring of desorption from adsorbents into the solution was also carried out in five replicates. Cu(II) concentration in the mine water was adjusted by the addition of CuSO_4_·5 H_2_O. The adsorption process was carried out under continuous stirring to ensure optimal contact between the aqueous phase and the adsorbent.

### 2.5. Atomic Absorption Spectrometry (AAS)

Metal concentrations during the adsorption experiments were measured by flame atomic absorption spectrometry using an AVANTA Σ instrument (GBC Scientific).

The Atomic Absorption Spectrometry (AAS) analysis was performed under the following conditions. First, the sample was prepared by filtration through a 5 µm filter to remove insoluble particles. The samples were then preserved with HNO_3_ to maintain a pH below 2. If necessary, microwave digestion was used for sample decomposition. For atomization, an acetylene flame with air as the oxidant was used, reaching a temperature of approximately 2000–3000 °C. The radiation source was a hollow cathode lamp specific to copper (Cu), with a wavelength of 324.8 nm and a slit width of 0.8 mm. Calibration was carried out using calibration solutions prepared in the same matrix as the samples. The calibration concentration range was selected according to the expected copper concentration and ranged from 0.25 to 7 mg/L. The quantitative determination was based on the linear relationship between absorbance and analyte concentration, according to the Beer–Lambert law.

### 2.6. Calculations

#### 2.6.1. Percentage of Metal Ion Removal Efficiency

The removal efficiency of metal ions was expressed as a percentage and determined using Equation (1) [53]:

(1) $$Ads.\;\%=\frac{\left(c_{0}-c_{e}\right)}{c_{o}}\times 100$$

where *c*_0_ denotes the initial concentration of the metal ion in solution (mg·dm^−3^), while *c_e_* represents its equilibrium concentration, or the concentration measured at a given time t (mg·dm^−3^).

#### 2.6.2. Adsorption Capacity

The adsorption capacity at equilibrium and at time t was calculated according to Equation (2) [53]:

(2) $$q_{e}=\frac{\left(c_{o}-c_{e}\right)\cdot V}{m}$$

where *c_o_* is the initial concentration of ions in solution (mg·dm^−3^), *c_e_* is the equilibrium concentration of ions in solution or the concentration of ions in solution at time t (mg·dm^−3^), *V* is the volume of solution (dm^−3^), *m* is the mass of adsorbent added (g), and *q_e_* is amount of adsorbed heavy metal per unit sorbent mass (mg·g^−1^).

#### 2.6.3. Langmuir Adsorption Isotherm

The Langmuir model describes adsorption as the formation of a single adsorbate layer on an energetically uniform surface composed of equivalent binding sites, with each site occupied by no more than one adsorbed species. This model is commonly applied to describe adsorption processes in which the adsorbate interacts with a limited number of active sites on the adsorbent surface. However, fitting the Langmuir model does not necessarily indicate a specific adsorption mechanism, such as chemisorption or electrostatic interactions, since the obtained parameters only describe the equilibrium adsorption behavior. The possible adsorption mechanism should therefore be evaluated based on additional analyses, such as kinetic modeling and surface characterization. The Langmuir isotherm is expressed by the relation [54]:

(3) $$q_{e}=\frac{q_{m}\cdot b\cdot c_{e}}{1+b\cdot c_{e}}$$

or in the linearized form [54]:

(4) $$\frac{c_{e}}{q_{e}}=\frac{1}{b\cdot q_{m}}+\frac{1}{q_{m}}\cdot c_{e}$$

where *q_m_* (mg·g^−1^) represents the theoretical maximum adsorption capacity corresponding to monolayer coverage, whereas *b* denotes the Langmuir equilibrium constant associated with the affinity between the adsorbent and adsorbate.

### 2.7. Statistical Analysis

The variability among the obtained samples was evaluated using two-way analysis of variance (ANOVA) to assess whether it could be attributed to random deviations or to the effects of the studied factors and their interaction. The null hypothesis assumed equality of the mean values of the individual groups. The test statistic was the F-ratio, defined as the ratio of two estimates of variance: the mean square of the factor effect (MS effect) and the mean square of the residual error (MS error). When the mean square associated with the tested effect exceeded the mean square error by a statistically significant margin, the null hypothesis was rejected, indicating that the evaluated factor had a significant influence on the observed differences among sample means. When statistically significant effects were detected, Duncan’s multiple range test was used as a post hoc method to identify specific differences between group means. The mean values, together with their 95% confidence intervals, are presented using box-and-whisker plots. All statistical analyses were performed using STATISTICA 14 software.

## 3. Results and Discussion

### 3.1. Point of Zero Charge and Its Relationship to Metal Adsorption

The determined point of zero charge (PZC) values differed considerably among the investigated adsorbents. For bentonite, the PZC was determined to be in the range of 6.1–6.2, whereas natural zeolite exhibited a substantially lower PZC of 1.8–2.0. Fly ash showed the highest PZC value, approximately 12.81.

These results provide important context for interpreting the adsorption experiments conducted at approximately neutral pH. At above PZC values, the adsorbent surface is generally characterized by a predominantly negative charge, which may promote electrostatic attraction of metal cations. This mechanism is particularly relevant for natural zeolite, for which the PZC was determined at very low values (1.8–2.0). Therefore, at the near-neutral pH applied in the adsorption experiments, its surface was likely negatively charged, which may have promoted the adsorption of Cu(II) and Cd(II) cations. This is consistent with the observed adsorption of metal ions by the zeolite.

Bentonite exhibited a PZC in the range of 6.1–6.2. Since the adsorption experiments were conducted at approximately neutral pH, which was close to or slightly above the PZC of bentonite, its surface can be assumed to have been partially or predominantly negatively charged. This may have contributed to the electrostatic attraction of Cu(II) and Cd(II) cations. However, because the PZC was close to neutral, electrostatic interactions in the case of bentonite may have been more sensitive to changes in pH than those in the case of zeolite.

In contrast, fly ash exhibited a very high PZC value (12.81). Therefore, at neutral pH, its surface was unlikely to exhibit the same degree of negative charge as the surfaces of zeolite and bentonite. Nevertheless, fly ash achieved a high Cu(II) removal efficiency, indicating that its adsorption behavior was not governed solely by electrostatic attraction between a negatively charged surface and metal cations. In the case of fly ash, other mechanisms, such as ion exchange, interactions with surface functional groups, and precipitation processes associated with its alkaline character and mineralogical composition, may have played an important role.

The high PZC value of fly ash (12.81) also indicates its strongly alkaline character. At elevated pH values, Cu(II) may undergo hydrolysis with the formation of hydroxo complexes, such as Cu(OH)_3_^−^ and Cu(OH)_4_^2−^, while under suitable conditions, the precipitation of Cu(OH)_2_ may also occur. These processes may significantly affect Cu(II) removal and represent possible additional mechanisms alongside surface adsorption. Considering the high PZC value of the fly ash and its mineralogical composition, particularly the relatively high content of portlandite (18 wt.%), it is therefore possible that alkaline conditions and the associated hydrolysis and precipitation processes contributed to the high Cu(II) removal efficiency. This may partly explain why fly ash achieved high Cu(II) removal efficiency despite its surface not being predominantly negatively charged at neutral according to its PZC value.

The PZC results therefore indicate that surface charge is an important factor in interpreting adsorption behavior, but it cannot be considered the sole mechanism governing metal removal efficiency. Differences in the PZC values of the investigated adsorbents, together with their chemical and mineralogical compositions, help explain the different adsorption behaviors of bentonite, zeolite, and fly ash. Thus, determination of the PZC provides a useful complementary parameter for interpreting possible electrostatic interactions between the adsorbents and metal ions.

### 3.2. Mineral Composition (XRPD)

Mineral composition of materials used for experiments is shown in Table 4.

**Figure 6:**
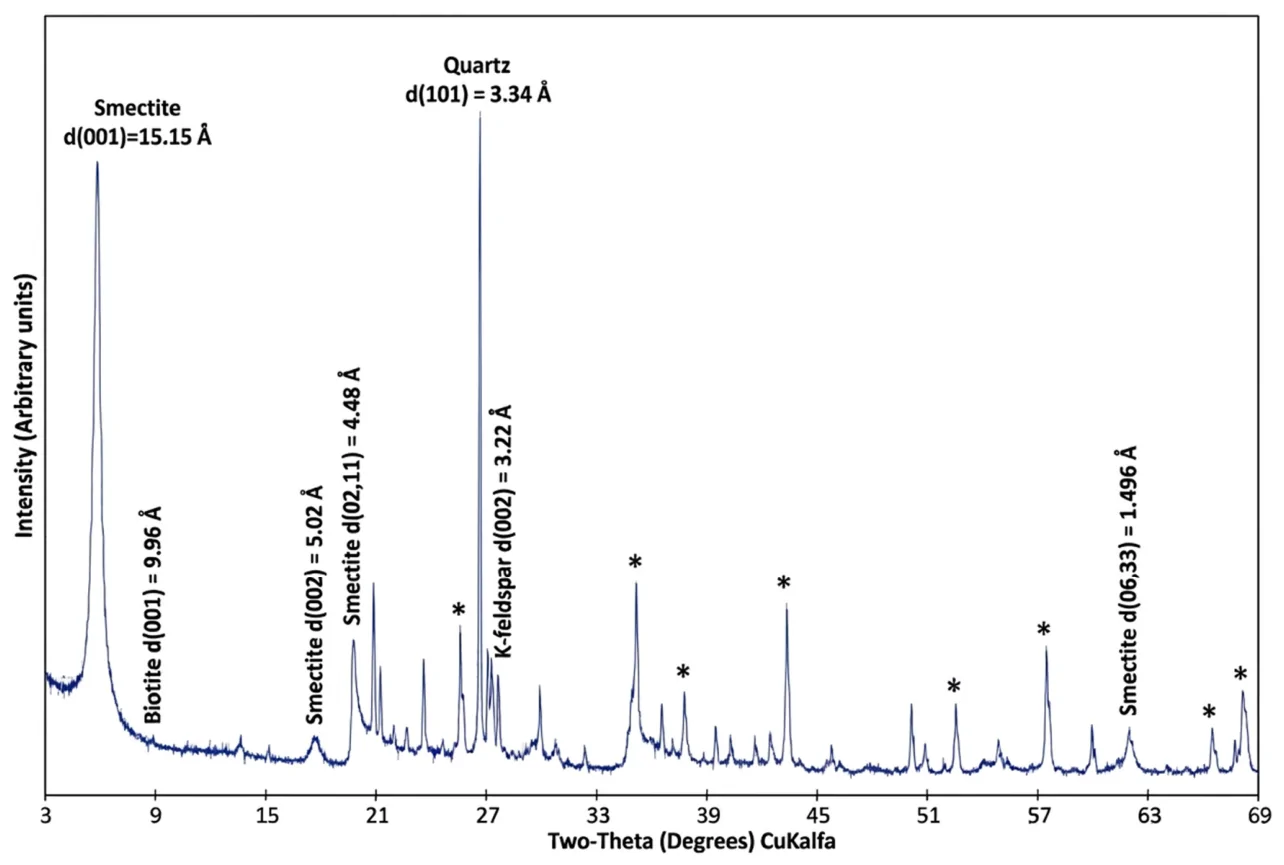
XRPD patterns of bentonite sample (Kopernica deposit). *—internal standard—corundum.

The XRPD pattern of the bentonite sample (Figure 6) indicated the presence of dioctahedral smectite (d(06,33) = 1.4964 Å) as the dominant mineral phase, accompanied by significant amounts of K-feldspars and quartz, together with minor traces of opal-CT and biotite. Quantitative phase analysis showed that the sample consisted of 57 wt.% smectite, 25 wt.% K-feldspars (orthoclase and sanidine), 16 wt.% quartz, and approximately 1 wt.% each of biotite and opal-CT.

**Figure 7:**
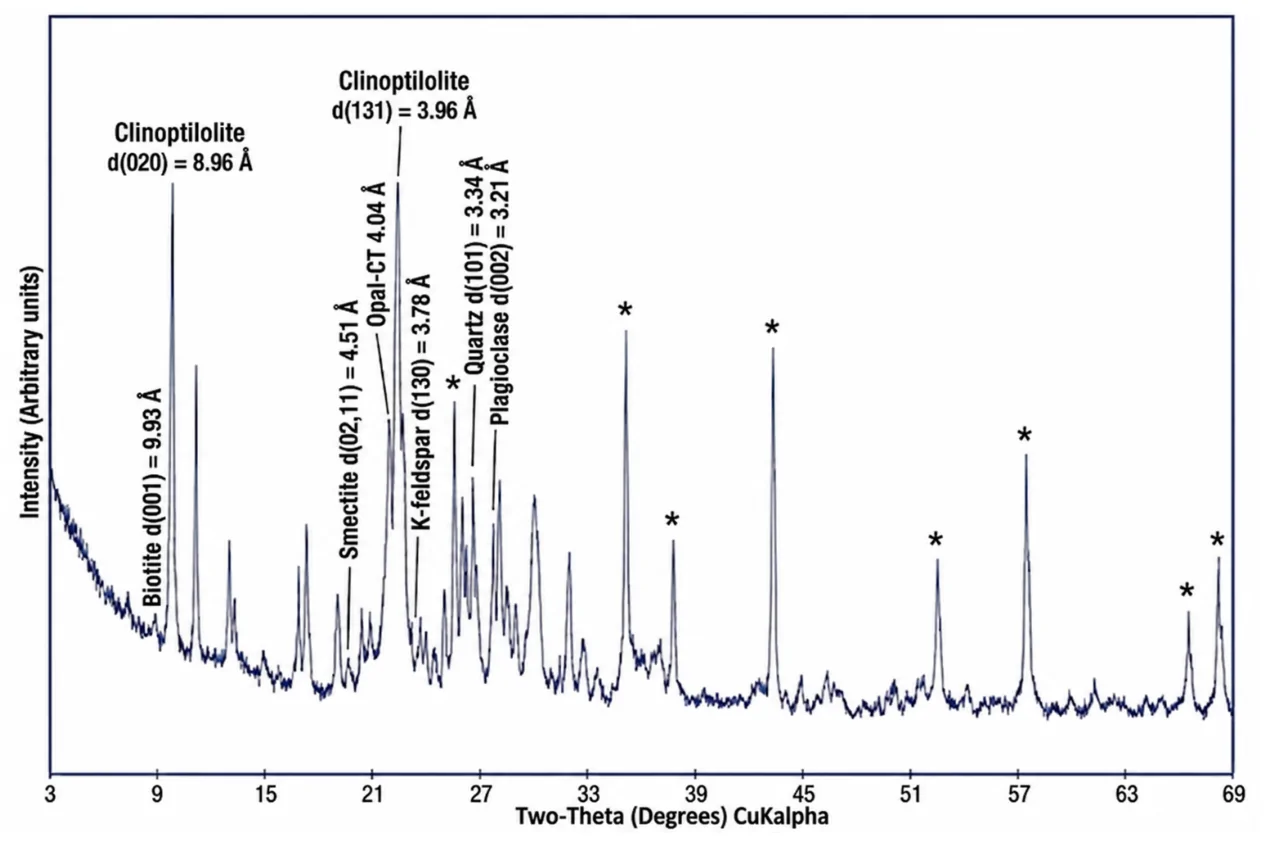
XRPD patterns of zeolite (Nižný Hrabovec deposit). *—internal standard—corundum.

The natural zeolite sample from the Nižný Hrabovec deposit (Figure 7) was dominated by clinoptilolite, which accounted for 76 wt.% of the material. Minor mineral phases included opal-CT (14 wt.%), plagioclase (3 wt.%), smectite (3 wt.%), K-feldspar (3 wt.%), and trace amounts of biotite and quartz (both <1 wt.%).

**Figure 8:**
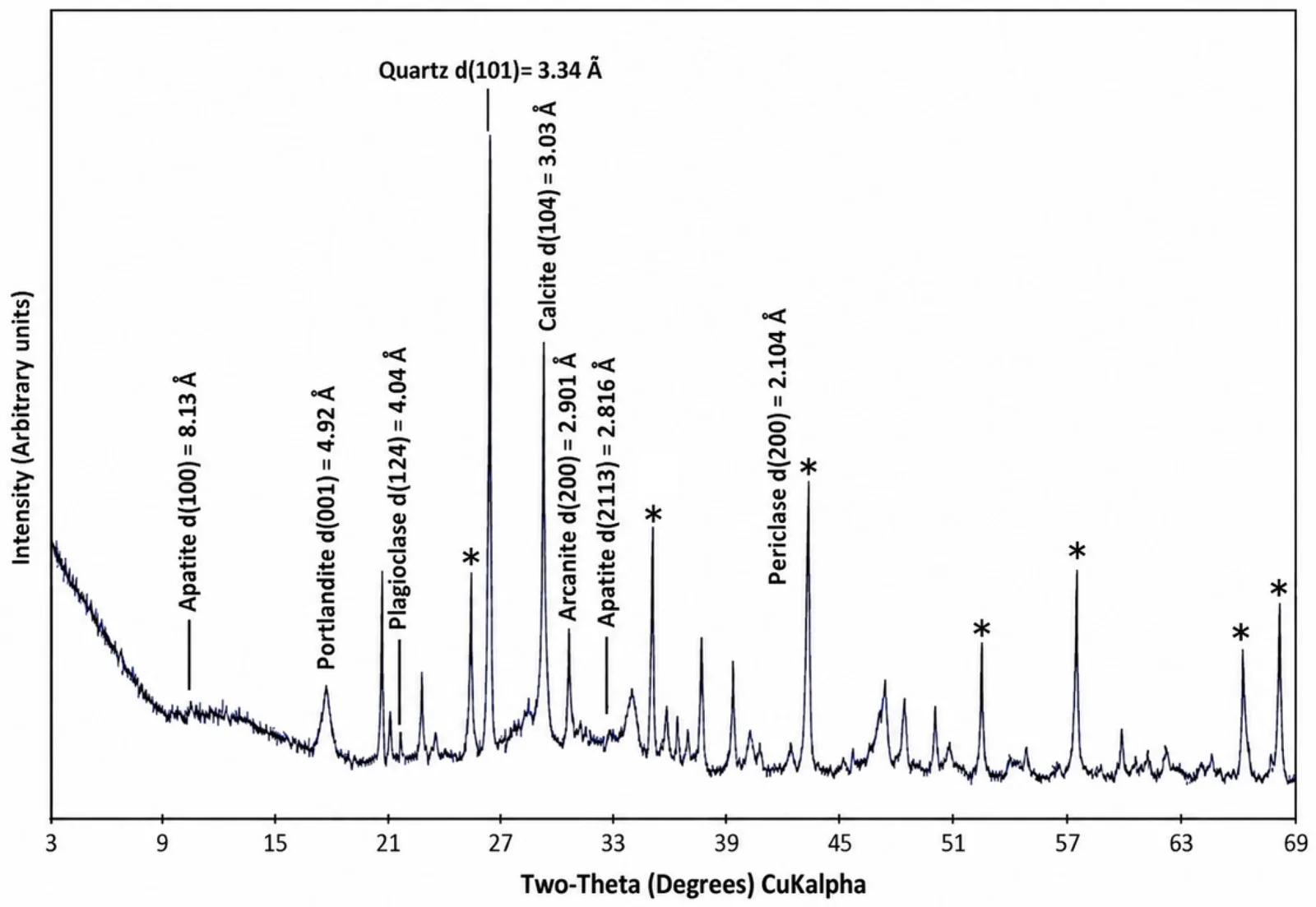
XRPD patterns of ash sample. *—internal standard—corundum.

The mineralogical composition of the fly ash sample (Figure 8) was determined by XRPD and comprised calcite (32 wt.%), portlandite (18 wt.%), quartz (18 wt.%), arcanite (16 wt.%), periclase (6 wt.%), and apatite (6 wt.%).

### 3.3. Relationship Between Mineralogical Composition and Adsorption Performance

The differences in the adsorption behavior of the investigated materials can be partially interpreted in relation to their distinct mineralogical compositions determined by XRPD analysis. Bentonite was predominantly composed of dioctahedral smectite (57 wt.%), whereas natural zeolite contained 76 wt.% clinoptilolite. These mineral phases represent potentially reactive components of the adsorbents and may contribute to metal ion sorption through ion exchange and interactions at mineral surfaces. In the case of bentonite, its adsorption behavior may be associated primarily with the presence of smectite, which is characterized by a high capacity for cation retention and cation exchange. For natural zeolite, the high proportion of clinoptilolite may promote the retention of metal cations through ion exchange within its aluminosilicate framework.

The XRPD results also demonstrate that neither bentonite nor zeolite represented mineralogically pure materials. In addition to smectite, bentonite contained 25 wt.% K-feldspars and 16 wt.% quartz, whereas the zeolite contained 14 wt.% opal-CT together with minor amounts of plagioclase, K-feldspar, and smectite. These mineral admixtures may affect the overall surface and chemical properties of the materials. Therefore, the observed adsorption behavior is likely to result from the combined contribution of several mineral and surface components. This interpretation is also consistent with the BET and ATR-FTIR results, which indicate that the adsorption performance cannot be explained by a single adsorbent parameter alone.

The fly ash exhibited a substantially different mineralogical composition, consisting mainly of calcite (32 wt.%), portlandite (18 wt.%), quartz (18 wt.%), and arcanite (16 wt.%), with smaller amounts of periclase and apatite. The high content of portlandite, together with the presence of calcite and periclase, may contribute to the alkaline character of the fly ash and its ability to modify the pH of the adsorption system. This characteristic may be important for metal removal, as an increase in pH can promote the hydrolysis of metal ions and, depending on the experimental conditions, the formation of poorly soluble hydroxide or other secondary phases. Therefore, in the case of Cu(II), the high removal efficiency observed for fly ash cannot necessarily be attributed solely to conventional surface adsorption, but may involve a combination of surface sorption, ion exchange, and precipitation processes associated with the alkaline mineralogical composition of the material.

This interpretation is further supported by the PZC results. While the zeolite exhibited a very low PZC (1.8–2.0) and bentonite showed a PZC of 6.1–6.2, fly ash exhibited a substantially higher PZC of 12.81. At the approximately neutral pH applied in the adsorption experiments, the high adsorption efficiency of fly ash therefore cannot be explained simply by electrostatic attraction between a negatively charged surface and Cu(II) cations. In this case, the mineralogical composition of the fly ash and the associated alkaline character are likely to have played a more important role.

Overall, the results indicate that mineralogical composition contributes substantially to the differences in the adsorption behavior of the investigated materials. Smectite in bentonite and clinoptilolite in natural zeolite provide potentially active sites for the retention of metal cations, particularly through ion exchange and surface interactions. In contrast, the mineral phases present in fly ash, particularly portlandite, calcite, and periclase, may contribute to metal removal through pH modification and subsequent precipitation processes. These findings indicate that mineralogical composition is an important factor when selecting adsorbents for applications involving the treatment of water contaminated with metal ions. However, the adsorption behavior cannot be attributed to a single mineral phase, but rather results from the combined effects of mineralogical composition, surface functional groups, specific surface area, and other physicochemical properties of the materials.

### 3.4. Effect of the Adsorbent and Cu(II) Addition on Cd(II) Concentration

Table 5 presents the results of the two-way analysis of variance, showing the interactive effect of copper content and adsorbent type on the investigated concentration of Cd(II).

Based on the two-way analysis of variance (Table 4), a highly significant statistical difference in Cd(II) concentration was confirmed, depending on both the type of adsorbent and the addition of Cu(II). For this reason, Duncan’s test was conducted to identify specific cases of statistical differences (Table 5).

**Figure 9:**
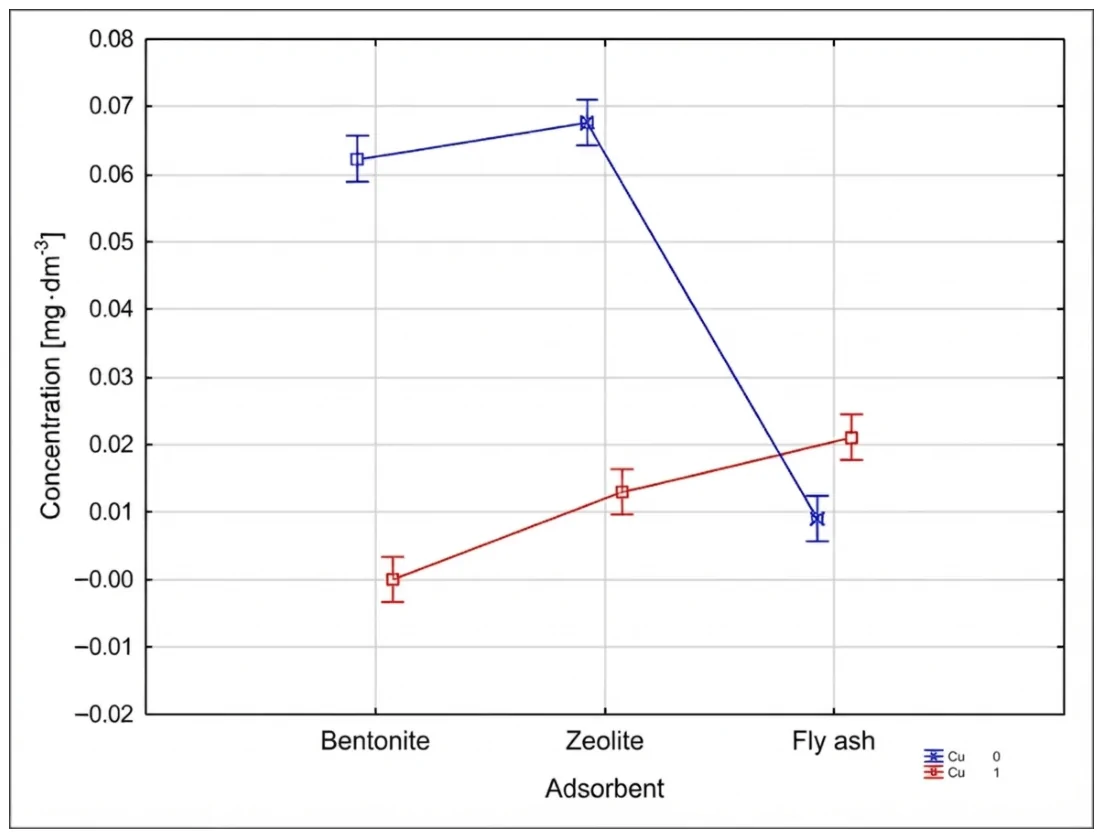
The 95% interval estimates for the mean value of the investigated concentration of Cd(II) for the three types of adsorbents, in the presence and absence of Cu.

Evaluation of Duncan’s test (Table 6) showed that the addition of Cu had a highly significant statistical effect on the concentration of Cd(II) during adsorption by bentonite, zeolite, and fly ash. The next step is to determine whether the addition of Cu caused an increase or decrease in Cd(II) concentration in neutral mine drainage during adsorption (Figure 9).

As shown in Figure 9, the addition of Cu during adsorption by bentonite and zeolite caused a decrease in Cd(II) concentration in the solution, indicating a positive adsorption effect. Both positive effects were evaluated as highly significant from a statistical perspective based on Duncan’s test results (Table 6). In contrast, the addition of Cu during adsorption by fly ash in neutral mine drainage caused an increase in Cd(II) concentration, indicating a negative adsorption effect (Figure 9). According to Duncan’s test (Table 6), this negative adsorption effect of Cu on Cd(II) concentration was confirmed as moderately significant.

Abu-El-Halawa and Zabin [55] reported efficient Cu(II) removal by zeolitic materials over a pH range of 3–7.3. In their study, the experimental system also involved a solution containing 12.7 g (0.075 mol) of diphenylamine and 1.80 g (0.075 mol) of NaH in 10 mL of anhydrous tetrahydrofuran. Ren et al. [56] likewise identified pH 6 as a favorable condition for Cu(II) uptake using a magnetic porous ferrospinel, with an adsorbent dose of 0.025 g MnFe_2_O_4_, while avoiding hydroxide precipitation and maintaining a sufficiently high adsorption capacity. In contrast, the addition of fly ash to mine water can markedly increase pH, which may influence the binding, mobility, and toxicity of Cd(II) in the aquatic environment. Because solution pH affects the surface charge of adsorbents, it represents one of the key factors controlling metal-ion adsorption [57]. In the study cited in [57], the amount of tannic acid immobilized on activated carbon was 1.69 mg·g^−1^. Other studies have also shown that metal-ion uptake may increase as pH rises, since strongly acidic conditions and elevated H^+^ concentrations can promote mineral dissolution and the release of ions into solution; a quartz-based soil adsorbent was applied at a dose of 3 g [58].

After evaluating the effect of Cu(II) addition on the behavior of adsorbents in neutral mine drainage, the percentage removal of Cd(II) (with and without Cu addition) and Cu(II) (with Cu addition) was assessed (Table 7). The results of the removal percentage of the hazardous element are important for evaluating the competitiveness of fly ash for Cd(II) adsorption compared to commercially used adsorbents, bentonite and zeolite.

The relatively high standard deviations observed for Cd adsorption on bentonite and zeolite in the absence of Cu(II) (Table 7) are associated with the negative removal efficiencies obtained under these conditions. These negative values indicate that Cd(II) was released from the adsorbent into the solution rather than removed, resulting in greater variability among replicate measurements. Therefore, the higher standard deviations are attributed to the variability of the adsorption/desorption process rather than to analytical determination.

The relatively high standard deviations observed for Cd(II) removal by bentonite and zeolite in the absence of Cu(II) (Table 6) are associated with the negative removal efficiencies obtained under these conditions. Negative removal efficiencies indicate that the Cd(II) concentration in the solution after the adsorption experiment was higher than the initial concentration, indicating a net release of Cd(II) from the adsorbent into the solution. Under these conditions, relatively small differences in the measured Cd(II) concentrations between replicates can result in substantial differences in the calculated removal efficiency. Therefore, the high standard deviations should be interpreted primarily in the context of the pronounced variability of the adsorption/desorption process and the calculation of removal efficiency, particularly at the low Cd(II) concentration level, rather than as direct evidence of poor analytical repeatability.

According to the results in Table 7, negative percentage removal was observed during the adsorption of Cd(II) with bentonite (−199.8%) and zeolite (−224.6%) without the addition of Cu. It is surprising that Cd(II) was released back into the solution, despite both adsorbents containing low concentrations of this element (bentonite—0.068 mg·kg^−1^; zeolite—0.547 mg·kg^−1^), as shown in Table 3. The addition of Cu caused an increase in the percentage removal of Cd(II) for both bentonite (from −199.8% to 100%) and zeolite (from −224.6 % to 38.4%). The highest negative percentage removal of Cd(II) was observed for zeolite, while the highest positive percentage removal was observed for bentonite (100%). Similar variability in Cd(II) removal has been reported for bentonite- and zeolite-based adsorption systems. The efficiency of Cd(II) adsorption strongly depends on mineral composition, cation exchange capacity and the presence of competing ions in solution. In neutral mine effluents, Cd(II) removal has also been shown to be influenced by the aqueous matrix, particularly by dissolved background ions and surface heterogeneity of the adsorbent. These factors may explain why Cu addition improved Cd(II) removal for bentonite, whereas zeolite showed a less favourable response under the tested conditions [17,59,60].

For Cd(II) adsorption from neutral mine drainage, a negative effect was observed for fly ash when Cu was added (percentage removal decreased from 57.297% to 0%). This indicates that fly ash is unsuitable for Cd(II) adsorption in the presence of Cu, as no Cd(II) removal occurred in this case. The highest efficiency of fly ash was observed for Cu(II) adsorption with Cu addition (98.8%). High Cu(II) removal efficiency with fly ash has been previously confirmed for unmodified fly ash in acidic mine drainage (99.69%) [61], for modified fly ash in aqueous solutions (98%) [62], and for unmodified fly ash in synthetic aqueous solutions (97.1%) [63]. These reported Cu(II) removal levels are comparable to the results obtained in this study [61,62,63]. Fly ash demonstrated competitiveness in the adsorption of both Cd(II) and Cu(II) compared to natural adsorbents (bentonite and zeolite).

Comparison of Cd(II) and Cu(II) adsorption revealed preferential adsorption of Cu(II) over Cd(II) when using fly ash, showing a substantial average difference in percentage removal (Table 6). Higher competitive ability of Cd(II) compared to Cu(II) has been previously reported for metal adsorption on sediments [64]. In that study [64], it was also observed that Cu(II) tended to displace Cd(II) from sediments. In contrast, in our study, no negative adsorption of Cd(II) by fly ash was observed. The preferential adsorption of Cu(II) over Cd(II) can be directly related to Pauling electronegativity and possibly the hydrated ionic radius of Cd(II) and Cu(II). Metals with higher Pauling electronegativity may exhibit greater adsorption capacity [65]. A larger hydrated ionic radius places the cationic charge further from the adsorbent surface, weakening the electrostatic interaction between the adsorbent and the cation [66]. In this study, the focus was on the actual ionic radius rather than the hydrated radius. According to [50], the adsorption of heavy metal ions increases as the ionic radius decreases. That study demonstrated that Cu(II) is adsorbed most effectively due to its smaller ionic radius, which facilitates penetration into the adsorbent pores. The Pauling electronegativity of Cd(II) is 1.7, with an ionic radius of 0.097 nm, while Cu(II) has a Pauling electronegativity of 1.9 and an ionic radius of 0.072 nm [67]. This indicates that Cu(II) has a higher electronegativity and a smaller ionic radius compared to Cd(II), confirming the positive effect of these properties on the superior adsorption of Cu(II) over Cd(II).

According to [57], the presence of other ions in solution affects the adsorption of a specific ion, as metal ions compete for available adsorption sites. Those with the highest ionic potential (charge-to-radius ratio) or greatest electronegativity are removed first, followed by ions with lower ionic potential or electronegativity if adsorption sites remain. During the co-adsorption of Cu(II) and Cd(II), Cu(II) and possibly other metals present in the neutral mine drainage preferentially occupied free adsorption sites, preventing Cd(II) from binding to the fly ash surface.

The zero-percentage removal of Cd(II) (with Cu(II) addition) using fly ash may have been caused by both the preferential adsorption of Cu(II) and the concurrent increase in pH. According to study [68] fly ash may exhibit highly variable adsorption efficiency depending on pH and mineral phase composition. Interestingly, despite this effect, the adsorption of Cd(II) without Cu addition and of Cu(II) was not disrupted.

**Figure 10:**
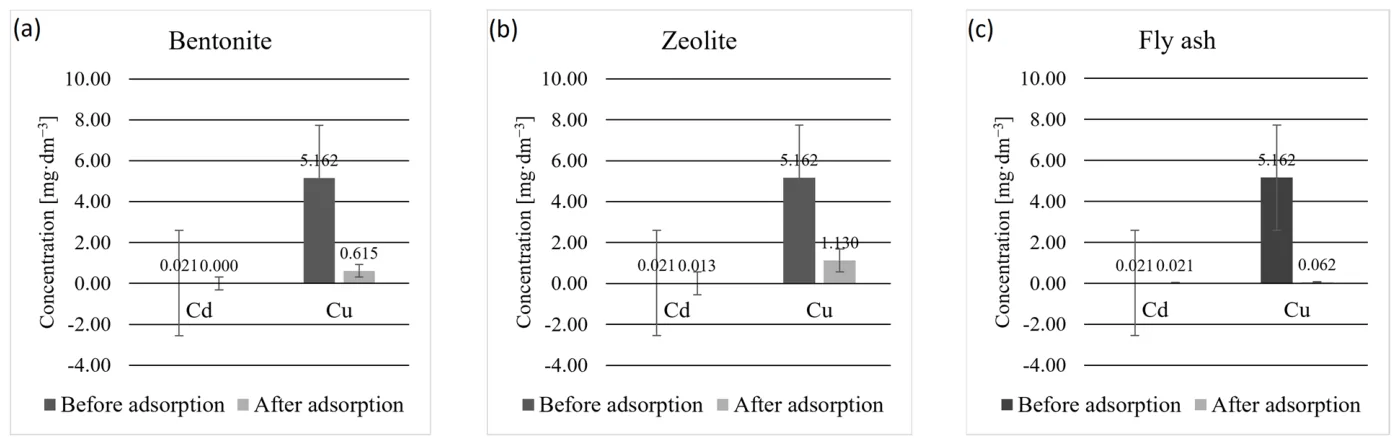
(**a**–**c**) Cd(II) and Cu(II) concentrations before and after adsorption in the presence of Cu; (**a**) bentonite, (**b**) zeolite, (**c**) fly ash.

The final objective of the study was to evaluate the desorption of Cd(II) and Cu(II) from the used adsorbents back into the mine drainage. Figure 10a–c provide an overview of the changes in Cd(II) and Cu(II) concentrations before and after adsorption by each adsorbent in the presence of Cu.

During adsorption with bentonite, zeolite, or ash, no desorption of Cd(II) or Cu(II) into the neutral mine drainage was observed (Figure 10a–c). As shown in Figure 10c, adsorption of Cd(II) by fly ash did not occur. This is likely due to preferential adsorption of Cu(II) on the functional sites of the ash, as a significant decrease in Cu(II) in the solution was observed in this case, or it could be caused by competition from ions of other elements present in the neutral mine drainage. The study by Zhenru Niu et al. [69] confirmed that a metal ion with a higher concentration exhibits stronger competitive ability, in accordance with the law of conservation of mass in chemical reactions. In addition to this reason, the preferential adsorption of metal ions on the adsorbent material may also be attributed to its mesoporous structure, which ensures efficient mass transport and strong binding of metal cations even at higher metal loadings [70].

### 3.5. Effect of the Adsorbent and Cu(II) Addition on Mn(II) Concentration

Table 8 presents the results of a two-way analysis of variance—the interactive effect of copper content and adsorbent type on the measured concentration of Mn(II).

Based on the two-way analysis of variance (Table 8), a highly significant statistical difference in Mn(II) concentration was confirmed, depending on both the type of adsorbent and the addition of Cu(II). For this reason, Duncan’s test was conducted to identify specific cases of statistical differences (Table 9).

**Figure 11:**
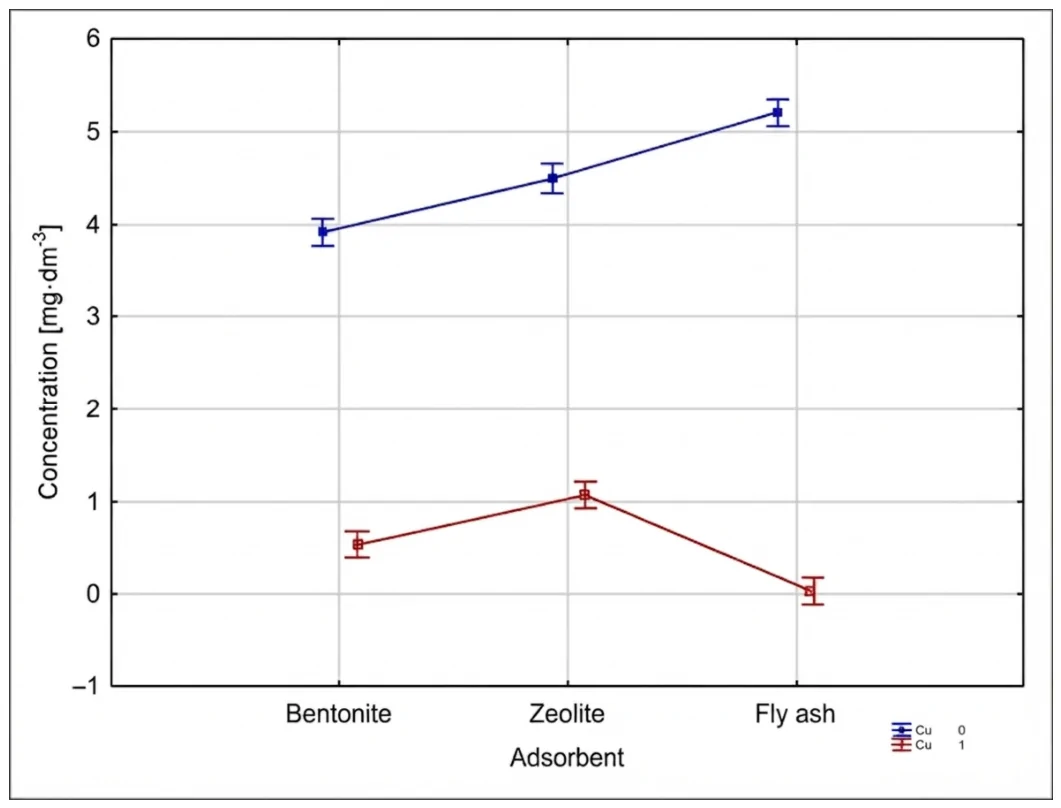
The 95% interval estimates for the mean value of the investigated concentration of Mn(II) for the three types of adsorbents, in the presence and absence of Cu.

Evaluation using Duncan’s test (Table 9) revealed that the addition of Cu had a highly significant effect on the concentration of Mn(II) during adsorption with bentonite, zeolite, and ash. The next step is to determine whether the addition of Cu caused an increase or decrease in Mn(II) concentration in the neutral mine drainage during adsorption (Figure 11).

According to Figure 11, the addition of Cu had a positive effect on adsorption with bentonite, zeolite, and ash, which was reflected by a decrease in Mn(II) concentrations in the neutral mine drainage. The greatest difference in Mn(II) concentration, with and without Cu addition, was observed when using fly ash. A similar trend has been reported for circumneutral mine drainage, where Mn(II) removal was strongly influenced by sorption reaction and surface controlled precipitation. Johnson and Hallberg described Mn as one of the more persistent dissolved metals in mine drainage systems, with removal depending strongly on mineral surface reactivity and water chemistry. Cravotta also reported that Mn retention under circumneutral conditions may significantly improve when sorption surfaces and geochemical conditions become more favourable. This may explain the positive effect of Cu addition observed for all adsorbents in the present study [71,72].

After evaluating the effect of Cu(II) addition on the behavior of the adsorbents in neutral mine drainage, the percentage removal of Mn(II) (with and without Cu addition) and Cu(II) (with Cu addition) was assessed (Table 10). The results of the removal percentages are important for evaluating the competitive capacity of ash for Mn(II) adsorption in comparison with already commercially used adsorbents—bentonite and zeolite.

The addition of Cu had a positive effect on the percentage removal of Mn(II) during adsorption with natural and waste adsorbents. In all three cases, Cu addition even promoted a shift from a negative to a positive Mn(II) removal percentage. The most pronounced enhancement of Mn(II) removal by Cu addition was observed with waste ash, where the removal increased from −40.042% to 99.248%. Comparable results have been reported for waste-derived sorbents used for mine water treatment. Industrial ash materials often provide alkaline mineral phases and heterogeneous adsorption sites that can enhance the removal of dissolved metal ions. In mine drainage systems, Mn removal is typically more difficult than Cu removal. Therefore the strong increase observed after Cu addition may indicate that Cu influenced the surface chemistry of ash and promoted more favorable adsorption conditions for Mn(II) [68,72]. When using fly ash, the highest removal percentages of the target element were observed for both Mn(II) and Cu(II) adsorption.

**Figure 12:**
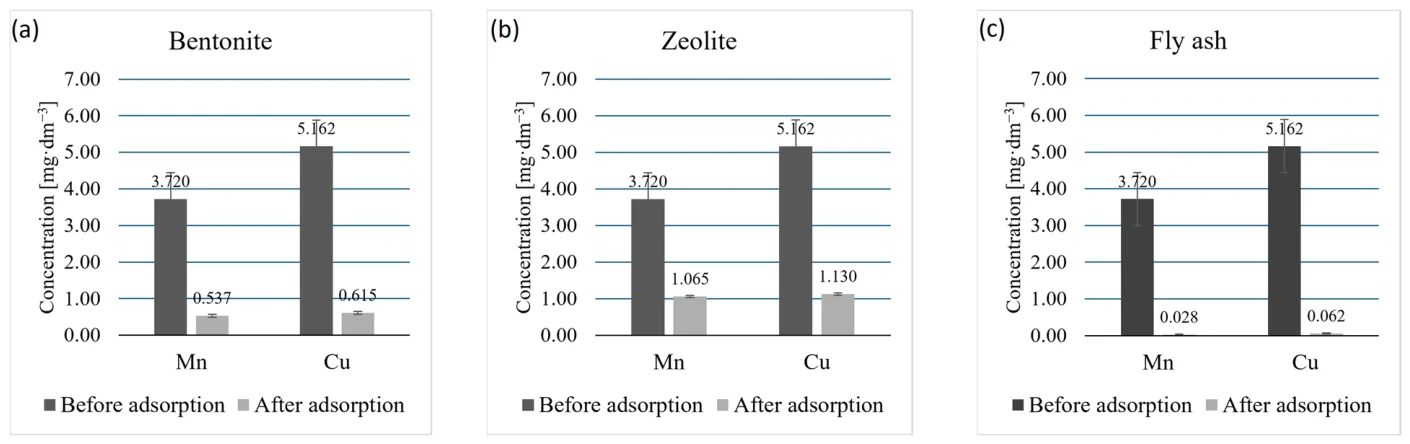
(**a**–**c**) Mn(II) and Cu(II) concentrations before and after adsorption in the presence of Cu; (**a**) bentonite, (**b**) zeolite, (**c**) fly ash.

The final objective of the study was to evaluate the desorption of Mn(II) and Cu(II) from the used adsorbents back into the mine drainage. Figure 12a–c provide an overview of the changes in Mn(II) and Cu(II) concentrations before and after adsorption by each adsorbent in the presence of Cu.

According to Figure 12a–c, no desorption of Mn(II) or Cu(II) into the neutral mine drainage was observed for any of the adsorbents used. From this, it can be concluded that an increased concentration of Cu(II) does not displace Mn(II) from the surface of bentonite, zeolite, or ash, despite the high concentrations of this element in bentonite, zeolite, and ash (Table 3). Similar behaviour has been reported for low-cost mineral sorbents, where metal ions remained strongly associated with active surface sites after adsorption. The stability observed in the present study is favourable for practical mine water treatment, as secondary release of adsorbed metals would reduce the long-term effectiveness of the treatment process [16,73].

### 3.6. Effect of the Adsorbent and Cu(II) Addition on Zn(II) Concentration

Table 11 presents the results of a two-way analysis of variance—the interactive effect of copper content and adsorbent type on the measured concentration of Zn(II).

Based on the two-way analysis of variance (Table 11), a highly significant difference in Zn(II) concentration was confirmed, depending on both the type of adsorbent and the addition of Cu(II). For this reason, Duncan’s test was conducted to identify specific cases of statistical differences (Table 12).

Evaluation using Duncan’s test (Table 11) revealed that the addition of Cu had a highly significant effect on the concentration of Zn(II) during adsorption with bentonite, zeolite, and ash. The next step is to determine whether the addition of Cu caused an increase or decrease in Zn(II) concentration in the neutral mine drainage during adsorption (Figure 7).

**Figure 13:**
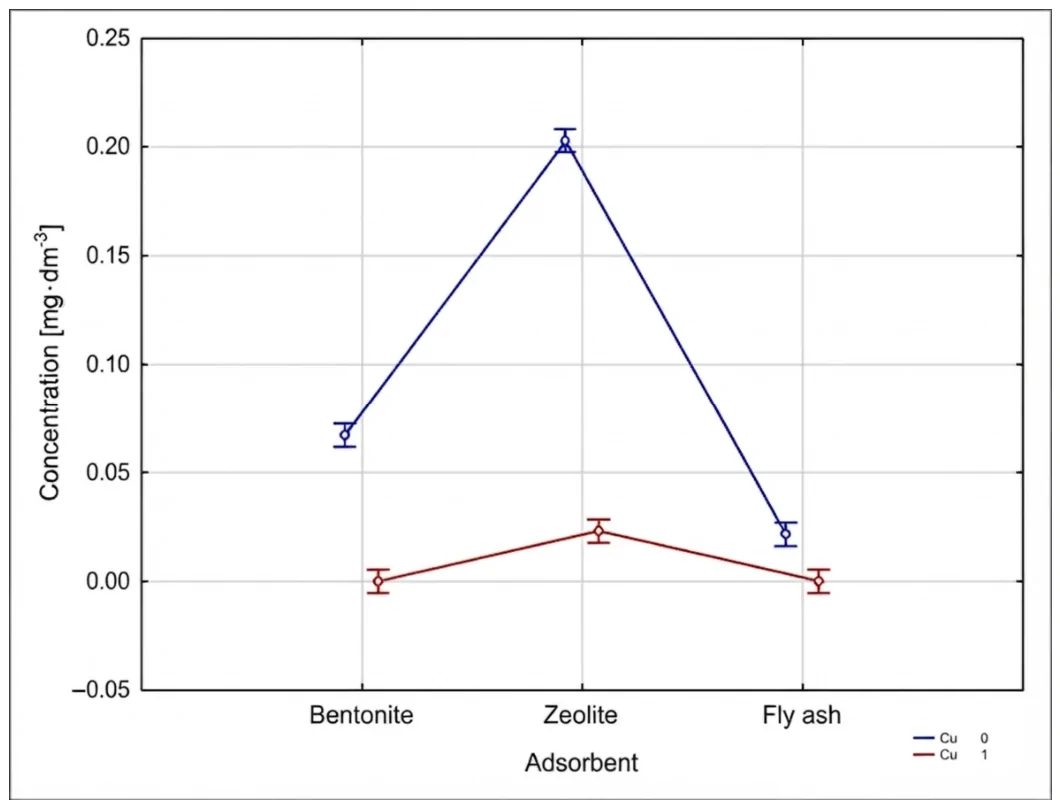
The 95% interval estimates for the mean value of the investigated concentration of Zn(II) for the three types of adsorbents, in the presence and absence of Cu.

According to Figure 13, the addition of Cu had a positive effect on adsorption with bentonite, zeolite, and ash, as reflected by a decrease in Zn(II) concentrations in the neutral mine drainage. The highest Zn(II) concentration in the neutral mine drainage was observed without Cu addition during adsorption with zeolite (approximately 20 mg·dm^−3^). This result is consistent with the study in [74], which demonstrates the synergistic effect of Cu(II) and Zn(II) during adsorption with bentonite. Comparable Zn(II) behavior has been reported for natural zeolites. Doula observed that simultaneous sorption of Zn(II) and Cu(II) on clinoptilolite was influenced by ion competition and adsorption site selectivity. Similar Zn removal from mine drainage using natural zeolite was described by Motsi et al., indicating that Zn(II) adsorption strongly depends on mineral composition and on the presence of coexisting metal ions. This may explain the lower Zn(II) concentrations observed after Cu addition in the present study [75,76].

After evaluating the effect of Cu(II) addition on the behavior of the adsorbents in neutral mine drainage, the percentage removal of Zn(II) (with and without Cu addition) and Cu(II) (with Cu addition) was assessed (Table 13). The removal percentage results are important for evaluating the competitive capacity of ash for Zn(II) adsorption in comparison with already commercially used adsorbents—bentonite and zeolite.

Based on the evaluation of the removal percentages (Table 13), fly ash can be considered competitive for the removal of both Zn(II) and Cu(II) from neutral mine drainage. According to the results in Table 13, no deterioration in adsorption was observed at increased Cu(II) concentrations. This finding contradicts the results of the study in [64], which reported that Zn(II) cations are significantly outcompeted for reactive surface sites on sediment particles by Cu(II) and are displaced from the sediments.

The low competition observed between Cu(II) and Zn(II) during adsorption can be discussed in terms of Pauling electronegativity and ionic radius. The ionic radius of Cu(II) is 0.072 nm, whereas that of Zn(II) is 0.074 nm [67], indicating that both cations have very similar ionic radii. This similarity likely contributed to the weak competition between Cu(II) and Zn(II) for adsorption sites on the used sorbents and prevented their desorption into the mine drainage, despite the higher Pauling electronegativity of Cu(II) (1.9) compared to Zn(II) (1.6) [67].

**Figure 14:**
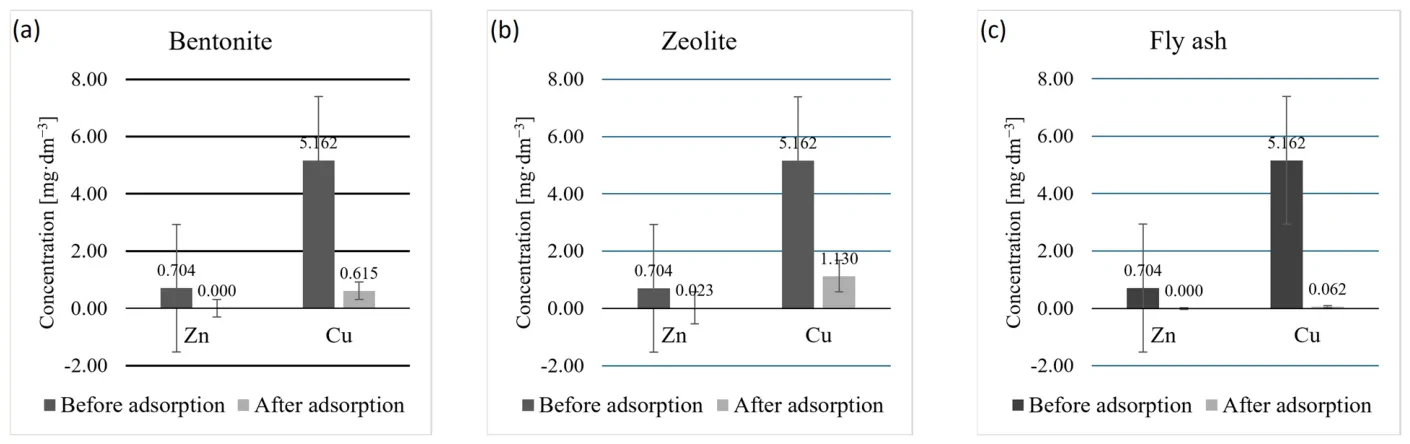
(**a**–**c**) Zn(II) and Cu(II) concentrations before and after adsorption in the presence of Cu; (**a**) bentonite, (**b**) zeolite, (**c**) fly ash.

The final objective of the study was to evaluate the desorption of Zn(II) and Cu(II) from the used adsorbents back into the mine drainage. Figure 14a–c provide an overview of the changes in Zn(II) and Cu(II) concentrations before and after adsorption by each adsorbent in the presence of Cu.

According to Figure 14a–c, no desorption of Zn(II) or Cu(II) into the solution was observed in any case. Based on the results presented in Figure 14a–c, it can be inferred that elevated Cu(II) concentrations do not inhibit Zn(II) adsorption or cause its desorption into the solution. This phenomenon is notable given the high concentrations of Cu(II) in bentonite and fly ash, and Zn(II) in bentonite, zeolite, and ash (Table 3). The absence of Zn(II) desorption indicates relatively stable retention of Zn on the investigated sorbents. Similar behavior has been described for mineral and low-cost adsorbents used for heavy metal removal, where Zn remained associated with active sorption sites after adsorption. Stable Zn retention is important for mine water treatment because secondary release of previously adsorbed metals may negatively affect long-term treatment efficiency [16,73].

### 3.7. Langmuir Adsorption Isotherm Analysis

Table 14 presents the parameters of the Langmuir adsorption isotherm depending on Cu addition.

The evaluation of Langmuir adsorption isotherm parameters revealed the highest *q_m_* values for Mn(II) adsorption using zeolite with Cu addition—0.8636 mg·g^−1^; biomass ash with Cu addition—0.7537 mg·g^−1^; and bentonite with Cu addition—0.7196 mg·g^−1^. Based on the coefficient of determination (*R*^2^), the Langmuir adsorption isotherm was found to be suitable in most cases. This suggests that, in our system, chemisorption or electrostatic adsorption occurred, where a monolayer is formed on the adsorbent surface and all active sites are considered equivalent. Although the Langmuir isotherm model showed a statistically significant fit to the experimental data for biomass ash across all investigated elements, the calculated maximum adsorption capacity should be interpreted with caution. Given the equilibrium pH of 9.4, the strong correlation with the Langmuir model cannot be attributed exclusively to pure surface adsorption. When comparing *q_m_* values, the underlying removal mechanisms must be differentiated. While bentonite and zeolite operate predominantly via surface adsorption and ion exchange, fly ash involves simultaneous adsorption and pH-induced chemical precipitation (caused by alkaline species leaching). Therefore, the highest *q_m_* observed for fly ash reflects its total removal capacity (combined adsorption and precipitation) rather than superior intrinsic adsorption performance alone. This phenomenon, in which precipitation mechanisms exhibit an apparent agreement with adsorption isotherms, is fully consistent with the findings reported by Sdiri and Higashi [51]. Cases where the Langmuir model was not suitable based on *R*^2^ values may be addressed by applying alternative isotherm models, such as the Freundlich or Temkin isotherms. Vengris et al. [77] described the adsorption of Cu(II) and other heavy metals by modified clay-based materials using the Langmuir model. For Cu(II), the reported maximum adsorption capacity (*q_m_*) was 83.3 mg·g^−1^ in the single-metal system and 80.3 mg·g^−1^ when three metal species were present simultaneously. When comparing their study with our results, significantly lower *q_m_* values were obtained in our case. This discrepancy may be attributed to the modification of adsorbents in the referenced study [77], as well as the reduction in *q_m_* values due to metal competition in neutral mine effluents. Indeed, Vengris et al. simulated wastewater using concentrated stock salt solutions [77].

Although considerably lower *q_m_* values were obtained in our study, it is important to emphasize that these results better reflect the real behavior of adsorbents in actual neutral mine waters. In such systems, due to cost constraints associated with adsorbent modification, the adsorption process may prove to be economically less favorable.

Similar findings have been reported in studies investigating the adsorption of heavy metals on natural aluminosilicate adsorbents. Several authors have shown that the Langmuir isotherm often provides a better fit for the adsorption of Cd(II), Zn(II), Cu(II), and Mn(II) on bentonite- and zeolite-based materials than the Freundlich model, particularly when adsorption occurs at a limited number of energetically similar active sites. This supports the assumption of predominantly monolayer adsorption on the adsorbent surface.

However, adsorption in real neutral mine waters represents a considerably more complex system than adsorption in synthetic single-metal solutions. The simultaneous presence of multiple dissolved metals may result in competition for active adsorption sites, which can reduce the apparent adsorption capacity and influence the agreement between experimental data and the Langmuir model. Such effects may partially explain the lower coefficients of determination observed in some adsorption systems investigated in this study. Nevertheless, the generally high *R*^2^ values obtained for most adsorbent–metal combinations indicate that the Langmuir model is suitable for describing the adsorption behaviour of the investigated systems and can provide useful information regarding the maximum adsorption capacity of the tested adsorbents [78,79].

### 3.8. Relationship Between BET Surface Area and Adsorption Performance

The BET specific surface area of the investigated adsorbents varied considerably, ranging from 4.1 m^2^·g^−1^ for bentonite to 33.2 m^2^·g^−1^ for natural zeolite, while fly ash exhibited an intermediate value of 15.1 m^2^·g^−1^ (Table 3). In general, a larger BET specific surface area provides a greater number of potential adsorption sites and is therefore expected to enhance adsorption capacity [80]. However, the results obtained in this study indicate that adsorption efficiency cannot be explained solely by the BET surface area. Although natural zeolite exhibited the highest BET specific surface area, fly ash achieved the highest Cu(II) removal efficiency. These findings suggest that adsorption was also strongly influenced by the physicochemical properties of the adsorbents, including surface functional groups [81,82], mineralogical composition [83], and the availability of active adsorption sites. Previous studies have shown that the mineralogical composition of adsorbents, which affects properties such as specific surface area and cation exchange capacity, plays a crucial role in the adsorption and desorption of heavy metals [83]. Similarly, adsorption performance does not necessarily correlate directly with the BET specific surface area, as surface chemistry and the nature of adsorption sites may have a greater influence on adsorption capacity [82]. Samghouli et al. [84] in their review state that for activated carbons used for adsorption of pharmaceutical pollutants, the adsorption capacity was not always correlated with the specific surface area. They point out the importance of surface functional groups and the chemical nature of the surface. The article also provides examples where a material with a lower specific surface area showed a higher adsorption capacity, and they state that the adsorption is influenced by a combination of specific surface area, porosity and surface functional groups.

### 3.9. Integrated Analysis of Adsorption Mechanisms Based on XRPD, PZC, BET, and ATR-FTIR Results

The adsorption performance and selectivity of the tested materials (bentonite, zeolite, and biomass fly ash) towards Cd(II), Mn(II), Zn(II), and Cu(II) in multi-component neutral mine drainage (NMD) cannot be attributed to a single physical property, but rather result from a complex synergy between mineral composition (XRPD), specific surface area (BET), surface charge potential (PZC), and active chemical functional groups (ATR-FTIR).

Although natural zeolite exhibited the highest BET surface area (33.2 m^2^·g^–1^) compared to biomass fly ash (15.1 m^2^·g^–1^) and bentonite (4.1 m^2^·g^–1^), the specific surface area was not the main governing factor determining maximum adsorption efficiency. Instead, surface chemistry and mineral phase interactions played a decisive role.

For bentonite and zeolite, the sorption mechanisms are predominantly driven by ion exchange and electrostatic surface complexation within their aluminosilicate structures. XRPD analysis confirmed that bentonite consists mainly of dioctahedral smectite (57 wt.%), while zeolite is dominated by clinoptilolite (76 wt.%). The low PZC of zeolite (1.8–2.0) ensures a strongly negatively charged surface at circum-neutral pH (7.4–7.7), promoting electrostatic attraction of dissolved metal cations. Bentonite’s PZC (6.1–6.2) also leaves its surface negatively charged under experimental conditions. The ATR-FTIR spectra for bentonite (Figure 2) and zeolite (Figure 3) show characteristic bands corresponding to (O–H) stretching/bending (3620 cm^−1^) and structural Si–O–Si/Si–O–Al vibrations (1018 cm^–1^). The slight band shifts (e.g., Si–O–Si bridge shift from 1018 to 1022 cm^–1^ in zeolite) post-adsorption reflect surface complexation and ion-exchange dynamics within the crystal lattice. When Cu(II) was introduced into the NMD system, Cd(II) removal efficiency for bentonite and zeolite increased significantly from negative values (desorption/release regime) to positive removals (100.0% and 38.4%, respectively), while Mn(II) and Zn(II) removals were also markedly enhanced. This behavior is attributed to synergistic ion exchange and surface activation induced by Cu(II) co-adsorption.

In contrast, biomass fly ash operates through a distinct multi-phase uptake mechanism involving surface complexation, ion exchange, and alkaline surface precipitation. XRPD reveals that fly ash comprises major mineral phases such as calcite (32 wt.%), portlandite (18 wt.%), quartz (18 wt.%), and arcanite (16 wt.%). The dissolution of alkaline oxides/hydroxides (specifically portlandite, Ca(OH)_2_) elevated the solution pH to 9.4. This coincides with the remarkably high PZC of fly ash (12.81), indicating that at neutral to slightly basic conditions, fly ash does not rely on net negative surface charge for cation capture. Instead, heavy metal removal occurs via hydrolysis, co-precipitation as metal hydroxides/carbonates, and interaction with silanol/aluminol surface groups. ATR-FTIR analysis of fly ash (Figure 4) supports this structural evolution: the disappearance of the isolated –OH band (3642 cm^–1^) and silica Si—O—Si band (1119 cm^–1^) after adsorption indicates the leaching of silica nanoparticles and chemical participation of hydroxyl active sites in metal immobilization.

Furthermore, the competitive selectivity observed in multi-component NMD—where Cu(II) completely suppressed Cd(II) adsorption on fly ash (0.0% removal with Cu vs. 57.3% without Cu) while enhancing Mn(II) (99.3%) and Zn(II) (100%) removal—can be explained by coordination chemistry and ionic parameters. Cu(II) possesses a higher Pauling electronegativity (1.9) and a smaller ionic radius (0.072 nm) compared to Cd(II) (1.7 and 0.097 nm, respectively), allowing Cu(II) to outcompete Cd(II) for available sorption/co-precipitation active sites. Zn(II), having a similar ionic radius (0.074 nm) to Cu(II), shows no competitive suppression.

Consequently, while the equilibrium adsorption data for fly ash show an excellent fit to the Langmuir isotherm *R*^2^ > 0.99 for Mn(II), Zn(II), and Cu(II), the calculated maximum capacities (*q_m_*) represent a combined contribution of chemisorption on heterogeneous functional sites and localized micro-precipitation. The complete absence of metal desorption post-treatment across all tested metals further confirms strong binding stability.

## 4. Conclusions

The present study demonstrated that the adsorption performance of low-cost adsorbents in neutral mine drainage strongly depends on competitive interactions among dissolved metals. The addition of Cu(II) significantly influenced the adsorption behaviour of Cd(II), Mn(II), and Zn(II), with the magnitude and direction of the effect varying according to the adsorbent type.

Among the investigated materials, biomass-derived fly ash exhibited the highest overall adsorption performance, achieving removal efficiencies of 98.8% for Cu(II), 99.3% for Mn(II), and complete removal of Zn(II). In contrast, Cu(II) completely suppressed Cd(II) adsorption on fly ash, reducing its removal efficiency from 57.3% to 0%, confirming the dominant competitive affinity of Cu(II) for the available adsorption sites. The preferential adsorption of Cu(II) can be explained by its higher electronegativity and smaller ionic radius compared with Cd(II), resulting in stronger interaction with the adsorbent surface.

ATR-FTIR analysis confirmed that adsorption was accompanied by changes in hydroxyl and aluminosilicate functional groups, indicating their participation in metal binding. In fly ash, the disappearance of the isolated hydroxyl band and changes in Si–O-related vibrations suggest structural modifications associated with adsorption, while shifts in characteristic bands observed for bentonite and zeolite indicate interactions between metal ions and surface functional groups.

The addition of biomass fly ash increased the equilibrium pH of neutral mine drainage to approximately 9.4. Under these alkaline conditions, heavy metal removal was likely governed not only by surface adsorption but also by partial surface precipitation or co-precipitation. Consequently, although the Langmuir model showed an excellent fit to the experimental data, the calculated maximum adsorption capacities for fly ash should be interpreted as representing the combined contribution of chemisorption and precipitation processes rather than pure monolayer adsorption.

No significant desorption of Cu(II), Mn(II), or Zn(II) was observed after adsorption, indicating stable immobilization of the retained metals and demonstrating the environmental suitability of the investigated adsorbents. The obtained results confirm that unmodified biomass fly ash represents a competitive and economically attractive alternative to natural bentonite and zeolite for the treatment of neutral mine drainage.

The lack of post-adsorption characterization and regeneration/reuse experiments represents a limitation of the present study and should be addressed in future work. Future research should focus on continuous-flow column experiments, regeneration and long-term stability of biomass fly ash, and detailed geochemical characterization of precipitation products to distinguish more precisely between adsorption and precipitation mechanisms under multicomponent conditions.

## Figures and Tables

**Table 1:** AAS operating conditions used for the determination of metals in mine water samples.

Metal	Analytical Method	Instrument	Atomization	Analytical Wavelength (nm)	Limit of Detection (LOD) (mg/L)	Sample Preparation
Cu	FAAS	AVANTA Σ (GBC Scientific)	Acetylene–air flame	324.8	0.005	Filtration (5 µm), acidification with HNO_3_ to pH < 2
Cd	FAAS	AVANTA Σ (GBC Scientific)	Acetylene–air flame	228.8	0.002	Filtration (5 µm), acidification with HNO_3_ to pH < 2
Mn	FAAS	AVANTA Σ (GBC Scientific)	Acetylene–air flame	279.5	0.003	Filtration (5 µm), acidification with HNO_3_ to pH < 2
Zn	FAAS	AVANTA Σ (GBC Scientific)	Acetylene–air flame	213.9	0.001	Filtration (5 µm), acidification with HNO_3_ to pH < 2

**Table 2:** Metal element concentrations and pH in NMD sample.

Source	Cu [μg/L]	Zn [μg/L]	Cd [μg/L]	Mn [μg/L]	Ni [μg/L]	Pb [μg/L]	Al [μg/L]	Fe [μg/L]	pH [–]
Slovak Regulation [22]	1.1–8.8	7.8–52	0.08–0.25	300	20	7.2	200	2000	–
Directive 2013/39/EU [23]	–	–	0.08–0.25 *	–	–	–	–	–	–
EPA—freshwater [24]	–	120	1.8	–	–	–	–	–	6.5–9
State Geological Institute of Dionysus Stur [25]	5	3435	14.8	1905	–	1.2	60	95	7.55
Measured value	**50–58**	**690–720**	**18–24**	**3700–3740**	7.5–9.1	1.5–2.1	145–159	12.0–12.6	7.4–7.7

* Cd limit depends on water hardness class. Elements exceeding Slovak legislative limits are highlighted in bold.

**Table 3:** Chemical composition, elemental analysis and BET characteristic of adsorbents.

**Adsorbent**	**Chemical Composition [%]**									
	**SiO_2_**	**Al_2_O_3_**	**Fe_2_O_3_**	**CaO**	**MgO**	**TiO_2_**	**Na_2_O**	**K_2_O**	**MnO**	
Bentonite [17]	60.18–73.8	11.56–24.90	2.15–3.39	1.23–2.17	1.09–3.29	0.11–0.20	0.29–0.91	0.42–1.18	0.02–0.06	
Zeolite	64.18–75.50	10.93–14.80	0.12–2.45	1.43–11.68	0.29–1.43	-	0.10–2.97	1.24–4.24	–	
Fly ash	–	–	–	–	–	–	–	–	–	
**Adsorbent**	**Elemental Analysis [mg·kg^−1^]**									
	Cu *	Mn *	Zn *	Cd *	**Pb**	**Si**	**As**	**Al**	**Sb**	**Fe**
Bentonite	2703	106.496	13.774	0.068	12.4	–	6.3	121.198	23.4	23.711
Zeolite	3.32	115.396	39.615	0.547	8.39	–	0.91	66.685	0.15	8.044
Fly ash	117	4771	1649	11.8	43.0	11,253	–	17,448	–	0.163
**Adsorbent**	**BET [m^2^·g^−1^]**									
Bentonite	4.1									
Zeolite	33.2									
Fly ash	15.1									

* Elements monitored during the adsorption process.

**Table 4:** Results of normalized quantitative XRPD analyses in weight %.

Sample	Method	Quartz	K-Feldspar	Plagioclase	Biotite	Clinoptilolite	Opal-CT	Arcanite	Calcite	Portlandite	Hydroxylapatite	Periclase	Smectite
Bentonite	RockJock	16	25		1		<1						57
Zeolite	RockJock	<1	3	3	<1	76	14						3
Ash	Rietveld	18		4				16	32	18	6	6	

Explanations: RockJock—Eberl, D.D. (2003) [49], Rietveld—Bruker [40].

**Table 5:** Results of the two-way analysis of variance—interactive effect of copper content and adsorbent type on the investigated concentration of Cd(II).

Effect	DF (Degree of Freedom)	SS (Sum of Squares)	MS (Mean Square)	F (F-Test)	P (*p*-Level)
Cu	1	0.009	0.009	727.354	0.000
Adsorbent	2	0.003	0.002	130.323	0.000
Cu × Adsorbent	2	0.008	0.004	331.091	0.000 ***
Error	24	0.000	0.000		
Total	29	0.021			

*** highly significant statistical difference *p* < 0.001. Values displayed as 0.000 represent calculated *p*-values < 0.0005 that were rounded to three decimal places.

**Table 6:** Duncan’s post hoc test for pairwise simultaneous comparisons of the investigated concentration of Cd(II).

	Cu	Adsorbent	{1} 0.060	{2} 0.068	{3} 0.084	{4} 0.101	{5} 0.065	{6} 0.000
1	0	B		0.024 **	0.000 ***	0.000 ***	0.000 ***	0.000 ***
2	0	Z	0.024 **		0.000 ***	0.000 ***	0.000 ***	0.000 ***
3	0	F	0.000 ***	0.000 ***		0.001 **	0.087	0.000 ***
4	1	B	0.000 ***	0.000 ***	0.001 **		0.000 ***	0.000 ***
5	1	Z	0.000 ***	0.000 ***	0.087	0.000 ***		0.002 **
6	1	F	0.000 ***	0.000 ***	0.000 ***	0.000 ***	0.002 **	

** moderately significant statistical difference 0.001 < *p* < 0.05; *** highly significant statistical difference *p* < 0.001. Values displayed as 0.000 represent calculated *p*-values < 0.0005 that were rounded to three decimal places.

**Table 7:** Percentage removal of Cd(II) under the applied conditions.

Element	Conditions	Average [%]	Standard Deviation [%]
Cd(II)	bentonite without Cu	−199.8	50.086
Cd(II)	bentonite with Cu	100.0	0.000
Cu(II)	bentonite with Cu	88.1	0.210
Cd(II)	zeolite without Cu	−224.6	36.705
Cd(II)	zeolite with Cu	38.4	8.055
Cu(II)	zeolite with Cu	78.1	1.281
Cd(II)	fly ash without Cu	57.3	8.743
Cd(II)	fly ash with Cu	0.0	0.000
Cu(II)	fly ash with Cu	98.8	0.054

**Table 8:** Results of the two-way analysis of variance—interactive effect of copper content and adsorbent type on the investigated concentration of Mn(II).

Effect	DF	SS	MS	F	*p*
Cu	1	119.628	119.628	4842.212	0.000
Adsorbent	2	1.626	0.813	32.914	0.000
Cu × Adsorbent	2	5.298	2.649	107.214	0.000 ***
Error	24	0.593	0.025		
Total	29	127.145			

*** highly significant statistical difference *p* < 0.001. Values displayed as 0.000 represent calculated *p*-values < 0.0005 that were rounded to three decimal places.

**Table 9:** Duncan’s post hoc test for pairwise simultaneous comparisons of the investigated concentration of Mn(II).

	Cu	Adsorbent	{1} 0.060	{2} 0.068	{3} 0.084	{4} 0.101	{5} 0.065	{6} 0.000
1	0	B		0.000 ***	0.000 ***	0.000 ***	0.000 ***	0.000 ***
2	0	Z	0.000 ***		0.000 ***	0.000 ***	0.000 ***	0.000 ***
3	0	F	0.000 ***	0.000 ***		0.000 ***	0.000 ***	0.000 ***
4	1	B	0.000 ***	0.000 ***	0.000 ***		0.000 ***	0.000 ***
5	1	Z	0.000 ***	0.000 ***	0.000 ***	0.000 ***		0.000 ***
6	1	F	0.000 ***	0.000 ***	0.000 ***	0.000 ***	0.000 ***	

*** highly significant statistical difference *p* < 0.001. Values displayed as 0.000 represent calculated *p*-values < 0.0005 that were rounded to three decimal places.

**Table 10:** Percentage removal of Mn(II) under the applied conditions.

Element	Conditions	Average [%]	Standard Deviation [%]
Mn(II)	bentonite without Cu	−5.1	0.268
Mn(II)	bentonite with Cu	85.6	0.187
Cu(II)	bentonite with Cu	88.1	0.210
Mn(II)	zeolite without Cu	−20.7	8.047
Mn(II)	zeolite with Cu	71.4	0.098
Cu(II)	zeolite with Cu	78.1	1.281
Mn(II)	fly ash without Cu	−40.0	5.400
Mn(II)	fly ash with Cu	99.3	0.108
Cu(II)	fly ash with Cu	98.8	0.054

**Table 11:** Results of the two-way analysis of variance—interactive effect of copper content and adsorbent type on the investigated concentration of Zn(II).

Effect	DF	SS	MS	F	*p*
Cu	1	0.060	0.060	1621.258	0.000
Adsorbent	2	0.058	0.029	779.405	0.000
Cu × Adsorbent	2	0.033	0.017	450.686	0.000 ***
Error	24	0.001	0.000		
Total	29	0.152			

*** highly significant statistical difference *p* < 0.001. Values displayed as 0.000 represent calculated *p*-values < 0.0005 that were rounded to three decimal places.

**Table 12:** Duncan’s post hoc test for pairwise simultaneous comparisons of the investigated concentration of Zn(II).

	Cd	Adsorbent	{1} 0.05960	{2} 0.06780	{3} 0.08360	{4} 0.10140	{5} 0.06480	{6} 0.0000
1	0	B		0.000 ***	0.000 ***	0.000 ***	0.000	0.000 ***
2	0	Z	0.000 ***		0.000 ***	0.000 ***	0.000	0.000 ***
3	0	F	0.000 ***	0.000 ***		0.000 ***	0.682	0.000 ***
4	1	B	0.000 ***	0.000	0.000 ***		0.000 ***	1.000
5	1	Z	0.000 ***	0.000 ***	0.682	0.000 ***		0.000 ***
6	1	F	0.000 ***	0.000 ***	0.000 ***	1.000	0.000 ***	

*** highly significant statistical difference *p* < 0.001. Values displayed as 0.000 represent calculated *p*-values < 0.0005 that were rounded to three decimal places.

**Table 13:** Percentage removal of Zn(II) under the applied conditions.

Element	Conditions	Average [%]	Standard Deviation [%]
Zn(II)	bentonite without Cu	90.5	0.452
Zn(II)	bentonite with Cu	100.0	0.000
Cu(II)	bentonite with Cu	88.1	0.210
Zn(II)	zeolite without Cu	71.1	2.315
Zn(II)	zeolite with Cu	96.8	0.245
Cu(II)	zeolite with Cu	78.1	1.281
Zn(II)	fly ash without Cu	97.0	0.608
Zn(II)	fly ash with Cu	100.0	0.000
Cu(II)	fly ash with Cu	98.8	0.054

**Table 14:** Parameters of the Langmuir adsorption isotherm depending on Cu addition.

Element	Conditions	Adsorbent	*q_m_* [mg·g^−1^]	Equation	Coefficient of Determination
Cd(II)	0	Bentonite	0.0111	y = 89.756x − 13.239	0.6048
		Zeolite	0.0369	y = 27.069x − 9.1005	0.0866
		Fly ash	0.0018	y = 557.14x − 1.1214	0.8128
	1	Bentonite	0.0032	y = 306.62x − 0.0579	0.9658
		Zeolite	0.001	y = 998.17x − 4.4464	0.7158
		Fly ash	8.69565 × 10^−5^	y = −11500x + 258.5	0.182
Mn(II)	0	Bentonite	0.005	y = 190.37x − 847.14	0.9832
		Zeolite	0.0276	y = 36.224x − 195.41	0.939
		Fly ash	0.1255	y = 7.9657x − 59.174	0.9824
	1	Bentonite	0.7196	y = 1.3896x + 0.0971	0.9921
		Zeolite	0.8636	y = 1.158x + 0.7723	0.9107
		Fly ash	0.7537	y = 1.3268x + 0.0008	0.9999
Zn(II)	0	Bentonite	0.1367	y = 7.3148x + 0.0363	0.7999
		Zeolite	0.0616	y = 16.231x − 1.2589	0.9476
		Fly ash	0.1355	y = 7.3806x − 0.001	0.9904
	1	Bentonite	0.1346	y = 7.427x − 0.0007	0.9983
		Zeolite	0.1713	y = 5.8382x + 0.0345	0.9959
		Fly ash	0.1346	y = 7.427x − 0.0007	0.9983
Cu(II)	0	Bentonite	−2.48515 × 10^−5^	y = −40239x + 2420.9	0.7116
		Zeolite	0.0032	y = 311.12x − 44.549	0.2427
		Fly ash	0.0028	y = 363.76x − 44.575	0.6551
	1	Bentonite	1.0502	y = 0.9522x + 0.0907	0.9562
		Zeolite	0.6827	y = 1.4647x − 0.2527	0.9945
		Fly ash	1.142	y = 0.8757x + 0.0065	0.9969

Note: 0—without Cu addition, 1—with Cu addition; B—bentonite, Z—zeolite, F—fly ash.

## Data Availability

The original contributions presented in the study are included in the article; further inquiries can be directed to the corresponding author.
